# Z-DNA is remodelled by ZBTB43 in prospermatogonia to safeguard the germline genome and epigenome

**DOI:** 10.1038/s41556-022-00941-9

**Published:** 2022-07-04

**Authors:** Yingying Meng, Guliang Wang, Hongjuan He, Kin H. Lau, Allison Hurt, Brianna J. Bixler, Andrea Parham, Seung-Gi Jin, Xingzhi Xu, Karen M. Vasquez, Gerd P. Pfeifer, Piroska E. Szabó

**Affiliations:** 1 Capital Normal University College of Life Science, Beijing, China; 2 Department of Epigenetics, Van Andel Institute, Grand Rapids, MI USA; 3 Division of Pharmacology and Toxicology, College of Pharmacy, The University of Texas at Austin, Austin, TX USA; 4 Bioinformatics and Biostatistics Core, Van Andel Institute, Grand Rapids, MI USA; 5 Van Andel Institute Graduate School, Grand Rapids, MI USA; 6 Guangdong Key Laboratory for Genome Stability & Disease Prevention and Carson International Cancer Center, Marshall Laboratory of Biomedical Engineering, Shenzhen University School of Medicine, Shenzhen, China; 7 Present Address: School of Life Science and Technology, Harbin Institute of Technology, Harbin, China

**Keywords:** Genomic instability, Spermatogenesis, DNA methylation

## Abstract

Mutagenic purine–pyrimidine repeats can adopt the left-handed Z-DNA conformation. DNA breaks at potential Z-DNA sites can lead to somatic mutations in cancer or to germline mutations that are transmitted to the next generation. It is not known whether any mechanism exists in the germ line to control Z-DNA structure and DNA breaks at purine–pyrimidine repeats. Here we provide genetic, epigenomic and biochemical evidence for the existence of a biological process that erases Z-DNA specifically in germ cells of the mouse male foetus. We show that a previously uncharacterized zinc finger protein, ZBTB43, binds to and removes Z-DNA, preventing the formation of DNA double-strand breaks. By removing Z-DNA, ZBTB43 also promotes de novo DNA methylation at CG-containing purine–pyrimidine repeats in prospermatogonia. Therefore, the genomic and epigenomic integrity of the species is safeguarded by remodelling DNA structure in the mammalian germ line during a critical window of germline epigenome reprogramming.

## Abstract (web summary)

Meng et al. show that ZBTB43 alters Z-DNA structures to prevent deleterious double-strand breaks and promote DNA methylation at purine–pyrimidine repeats in the mouse germ line.

## Main

In addition to the right-handed B-DNA helix, DNA can be detected in alternative forms including a left-handed double helix, called Z-DNA^1–3^. Purine–pyrimidine repeats (PPRs), which can adopt the Z-DNA conformation and induce DNA double-strand breaks (DSBs), are risk factors for genomic re-arrangements and cancer^4,5^. In mammalian cells, Z-DNA structures formed at (CG)*n* repeats are recognized and cleaved by ERCC1-XPF and other enzymes, producing DSBs around the structure itself. When DSBs are repaired through error-generating repair events, such as the non-homologous end joining (NHEJ) pathway, large-scale genomic re-arrangements, deletions and translocations occur in a replication-independent manner^6,7^. Importantly, if such mutagenic events are not controlled in the germ line, they may endanger the genome integrity of the species. It is not known whether the germ line has any means of curbing mutations at PPRs, or whether the Z-DNA structure is controlled in germ cells. Here we show that Z-DNA is remodelled in the mammalian germ line.

ZBTB43 is a member of the Broad-complex, Tramtrack and Bric-à-brac (BTB) zinc-finger-domain-containing protein (ZBTB) family^8^. Each ZBTB protein contains C2H2 zinc fingers (ZFs), which are responsible for recognizing and binding specific DNA sequences, and the BTB domain, which mediates protein–protein interactions. ZBTB proteins control various developmental and cellular processes^9^. In this Article, we characterize ZBTB43 as a Z-DNA-binding protein that is required for removing Z-DNA during a critical window of global epigenome remodelling in mouse prospermatogonia.

## Results

### Regulation of Z-DNA levels in the germ line

**Fig. 1:**
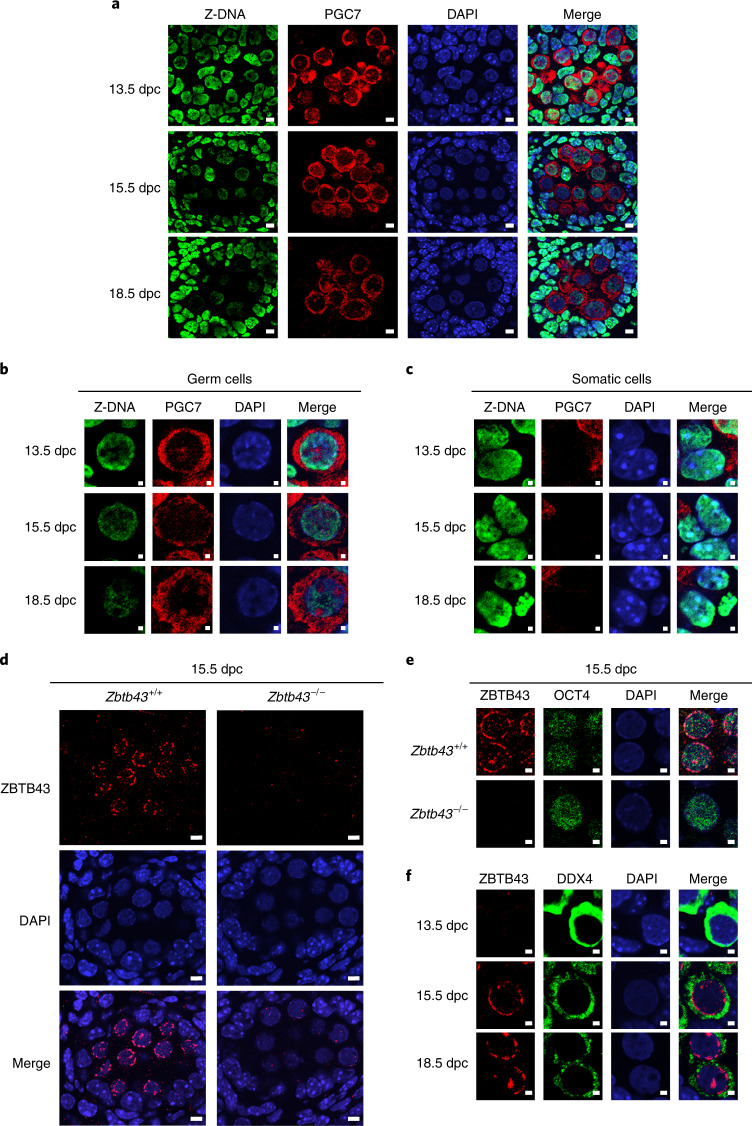
Natural removal of Z-DNA in germ cells of the mouse foetus. Immunohistochemistry images of testis samples are shown from male mouse foetuses. **a**, Z-DNA antibody staining (green) is diminishing in germ cells, as identified by PGC7 staining (red) at 13.5, 15.5 and 18.5 dpc. Scale bars, 5 µm. **b**,**c**, Loss of Z-DNA is specific to the germ cells in the foetal testis. Immunohistochemistry is shown for foetal testis sections at 13.5, 15.5 and 18.5 dpc timepoints, using Z-DNA antibody (green), germ cell marker (PGC7) and DAPI counterstain. Merged images are displayed on the right. Prospermatogonia (**b**) exhibit loss of Z-DNA. Somatic cells (**c**) maintain Z-DNA. Scale bars, 1 µm. **d**, ZBTB43 protein is detected in germ cells inside the testicular cords of the wild-type but not in the *Zbtb43*^−/−^ mutant testis at 15.5 dpc. Note the typical weak and diffuse DAPI staining of germ cell nuclei. The surrounding Sertoli cells, which exhibit stronger DAPI staining, are negative for ZBTB43 staining. Scale bars, 5 µm. **e**, Double staining of 15.5 dpc germ cells with ZBTB43 and germ cell marker OCT4 antibodies is shown in testis samples at 15.5 dpc. Scale bars, 2 µm. **f**, Double staining of testis samples using the ZBTB43 and germ cell marker DDX4 antibodies is shown at the foetal days as marked. Scale bars, 2 µm. The results shown represent three independent immunostaining experiments using four biologically independent testis samples per experiment (**a**–**c**), five independent experiments using three biologically independent testis samples per experiment (**d**), one immunostaining experiment using two biologically independent testis samples (**e**) and two independent immunostaining experiments using two biologically independent testis samples per experiment (**f**).

To detect Z-DNA in prospermatogonia globally, we carried out immunostaining of mouse foetal testis samples using a monoclonal anti Z-DNA antibody, Z22 (ref. ^10^). Z-DNA staining declines as a function of gestational age (Fig. 1a). This reduction was found in the PGC7-positive germ cells in the testicular cords at 15.5 days post coitum (dpc) but not in somatic cells of the testis (Fig. 1b,c). This finding reveals the existence of a process that reduces Z-DNA levels specifically in the germ cells at the time of major epigenome remodelling in foetal mouse germ cells^11–14^.

### *Zbtb43* RNA is highly expressed in prospermatogonia

**Extended Data Fig. 1:**
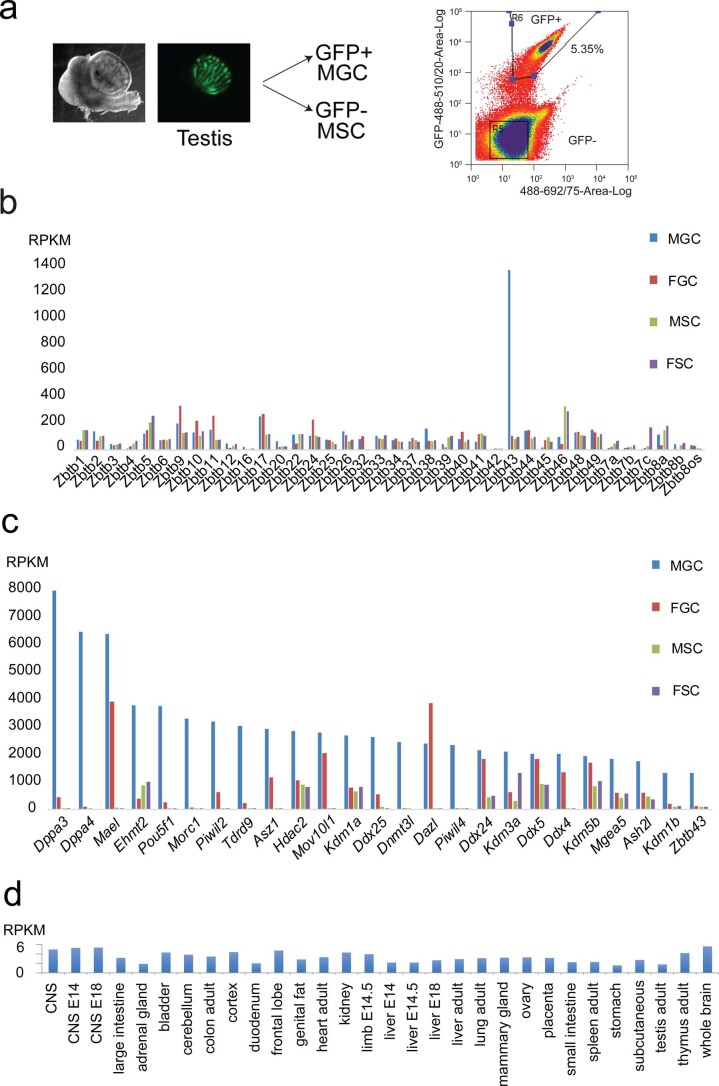
*Zbtb43* is specific to fetal male germ cells that remodel Z-DNA. (**a**) The fetal testis and mesonephros are shown in transmitted light to the left. Testicular cords are visualized by transgenic EGFP expression to the right. EGFP expression in the TgOG2 mouse line^15^ allows germ cell isolation by FACS. MGC: male germ cell (EGFP positive); MSC: male somatic cell (EGFP negative). FACS gating strategy is shown. R6: EGFP+, R5: EGFP-. (**b**) RNA-seq results are displayed in purified male germ cells (MGC), female germ cells (FGC) and somatic cells of male and female gonads (MSC and FSC) at 15.5 dpc^14^ (GSE46953). Transcript levels of *Zbtb43* are plotted among other *Zbtb* family members. (**c**) *Zbtb43* expression is depicted among some of the most highly expressed transcripts (apart from ribosomal proteins, elongation complex members or chaperons) in purified MGC, FGC, MSC, and FSC at 15.5 dpc. (**d**) *Zbtb43* expression is shown for mouse organs outside of the gonads. GEO Profiles: GDS661/116970_at from #BioProject PRJNA66167.

To understand the epigenome remodelling processes that take place in foetal male germ cells (MGCs) at 15.5 dpc, we focused on genes encoding transcription factors and epigenome remodellers. According to our RNA-seq data^14^ in purified foetal germ cells^15^ (Extended Data Fig. 1a), the transcription of *Zbtb43* was by far the highest among all *Zbtbs* in prospermatogonia at 15.5 dpc (Extended Data Fig. 1b). *Zbtb43* transcript levels were at over 1,300 reads per 1 kb, per million reads (RPKM) in MGCs, but 10–20-fold lower in the female germ cells (FGC) and somatic cells of the foetal gonads. *Zbtb43* was one of the most highly expressed genes in MGC, among important genes that carry out germ cell functions, such as *Dppa3*, *Mael*, *Dnmt3l*, *Pou5f1*, *Piwil2*, *Piwil4*, *Asz1* and *Dazl* (Extended Data Fig. 1c). Notably, in adult and foetal mouse organs the transcript level of *Zbtb43* is below 6 RPKM, many orders of magnitude lower than in MGC (Extended Data Fig. 1d). These data suggested that the ZBTB43 protein may carry out an important role in prospermatogonia.

### Temporally specific ZBTB43 expression in prospermatogonia

**Extended Data Fig. 2:**
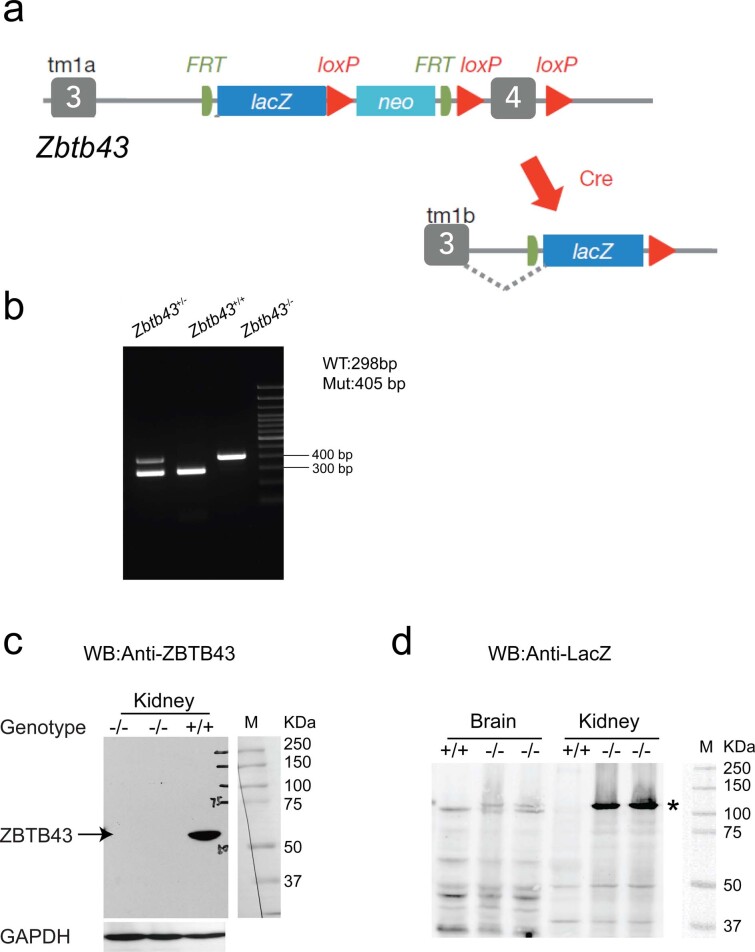
Validation of *Zbtb43*^-/-^ knockout mice. (**a**) Knockout strategy. Mice carrying the Zbtb43^tm1b(KOMP)Mbp^ allele. A *LacZ* cassette replaced exon 4 of the *Zbtb43* gene. (**b**) PCR genotyping from tail DNA to distinguish *Zbtb43*^+/+^, *Zbtb43*^+/-^ and *Zbtb43*^-/-^ pups at weaning. Data shown represent three independent experiments. (**c**) Western blot in adult kidney samples using the anti-ZBTB43 antibody and GAPDH loading control. (**d**) Western blot in adult kidney and brain samples using the anti-LacZ antibody. Data shown represent two independent experiments in c and d. Source data

To visualize ZBTB43 protein localization in prospermatogonia, we carried out immunostaining of 15.5 dpc foetal testes with the anti-ZBTB43 antibody. We found specific staining of the MGC in the perinucleus and nuclear speckles. (Fig. 1d). In the testicular cords, ZBTB43 protein expression was specific to germ cells, identified by positive staining with anti-OCT4 (Fig. 1e) or anti-DDX4 antibodies (Fig. 1f). Whereas wild-type prospermatogonia exhibited strong ZBTB43 staining at 15.5 dpc, the ZBTB43 signal was absent in the germ cells of a *Zbtb43*^−/−^ mutant foetus (Fig. 1d,e and Extended Data Fig. 2). To determine the timing of ZBTB43 expression, we stained testis sections with antibodies against ZBTB43 and the germ cell marker DDX4 at 13.5, 15.5 and 18.5 dpc. ZBTB43 expression was switched on in MGC between 13.5 and 15.5 dpc (Fig. 1f), coincident with Z-DNA elimination (Fig. 1a), which led us to explore whether these events were connected.

### ZBTB43 binds PPRs

**Fig. 2:**
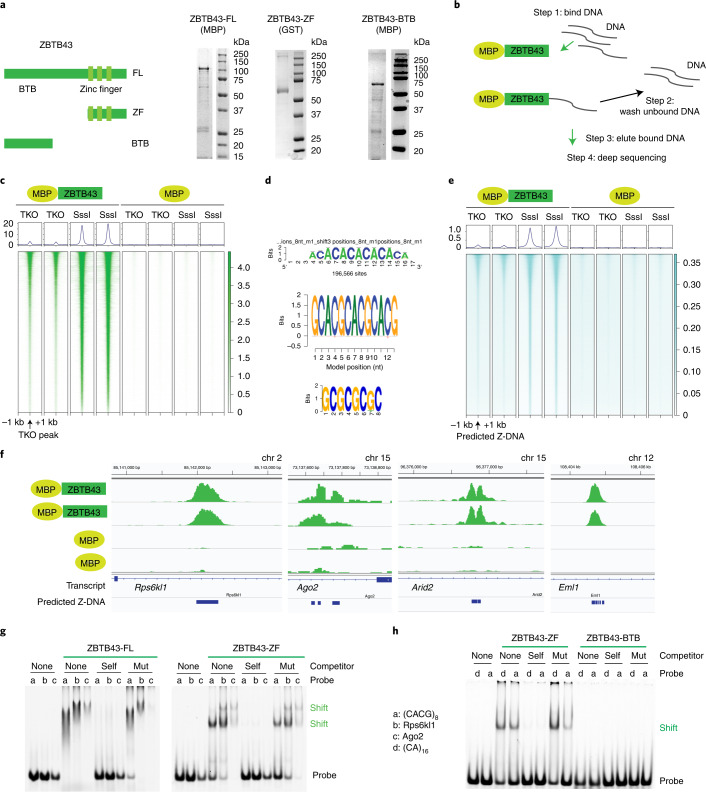
ZBTB43 is a DNA binding protein with affinity to PPRs. **a**, Structure of the ZBTB43 protein. Location of the BTB domain and the three ZF domains are indicated. The full-length protein (ZBTB43-FL), its ZF domain (ZBTB43-ZF) and its BTB domain (ZBTB43-BTB) were purified, as shown in the protein gels to the right. Protein purification and protein gel testing was done twice for ZBTB-FL and ZBTB-ZF and once for ZBTB43-BTB. **b**, Outline of the affinity sequencing experiment. MBP tag was used for the capture. **c**, ZBTB43 protein has affinity to methylated and unmethylated genomic DNA. Heatmap analysis of MBP-ZBTB43-FL binding is shown to genomic DNA, either DNMT-TKO ES cell DNA^16^ or DNA fully methylated by SssI bacterial CpG methyltransferase centred at the TKO DNA peaks. Background level binding by MBP is shown on the right. **d**, Consensus binding sequences of the affinity peaks in TKO determined by RSAT^43^ (top), significance 4.5e^−13^, BaMM^44^ (middle), dataset performance 0.096, motif performance 0.84, and MEME^45^ (bottom), significance 4.7e^−2775^. **e**, Heatmap shows the match between ZBTB43 affinity binding and predicted Z-DNA sites^17^. **f**, IGV browser images of selected specific MBP-ZBTB43 peaks are shown in TKO DNA samples at four genomic regions. Control samples show the background of MBP capture. The tracks for transcripts and predicted Z-DNA are displayed at the bottom. The affinity-sequencing results shown represent two independent biological replicates in **c**, **e** and **f**. **g**, EMSA confirm the binding of ZBTB43-FL or ZBTB43-ZF to the regions detected by affinity sequencing. The FAM-labelled probes (CACG)_8_, *Rps6kl1*, and *Ago2*, marked as a, b, and c, respectively, were competed out of the complexes by 100-fold excess of specific (Self) but not by the mutant (Mut) cold competitor. (**h)** EMSA confirms the binding of ZBTB43-ZF to the consensus PPR sequences. The FAM-labelled probes (CA)_16_ and (CACG)_8_, marked as d and a, respectively, resulted in specific shift; they were competed out of the complexes by 100-fold excess of specific (Self) but not by the mutant (Mut) cold competitor. The BTB domain of ZBTB43 (ZBTB43-BTB) lacked binding activity. Data shown represent three independent experiments in panels g and h. Source data

**Extended Data Fig. 3:**
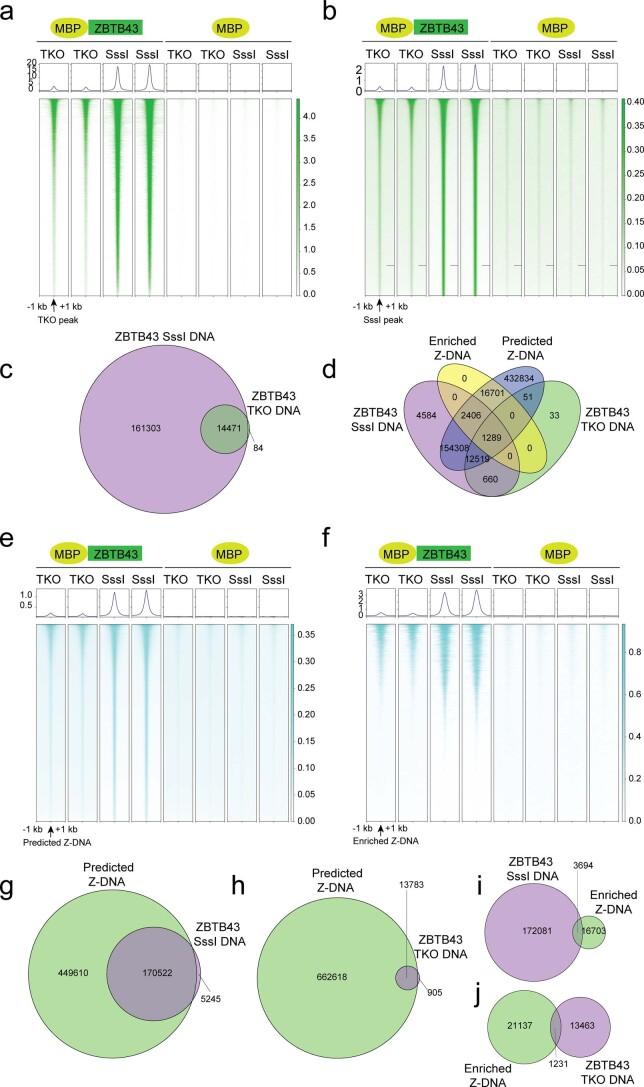
ZBTB43 protein has affinity to methylated and unmethylated genomic DNA and to predicted Z-DNA-forming sequences. (**a-b**) Heatmap analysis is shown of MBP-ZBTB43 (FL) binding intensities to genomic DNA, either *Dnmt1/Dnmt3a/Dnmt3b* triple knockout (TKO) ES cell DNA^16^ or DNA fully methylated by SssI bacterial CpG methyltransferase (SssI) centered at the TKO DNA peaks (**a**) and at the SssI peaks (**b**). Background level binding by MBP is included to the right. (**c**) Venn diagram shows the relationship between ZBTB43 affinity peaks mapped in SssI and TKO DNA. (**d**) Venn diagram shows the relationship between ZBTB43 affinity peaks mapped in SssI DNA, TKO DNA, and predicted Z-DNA sites^17^ or enriched Z-DNA sites in activated B-cells^18^. (**e-f**) Heatmaps show the match between ZBTB43 affinity binding peaks and Z-DNA sites. The affinity intensities are centered at predicted Z-DNA sites (**e**) and enriched Z-DNA sites mapped in activated B-cells (**f**). (**g-h**) Venn diagrams displays the relationship between predicted Z-DNA locations and ZBTB43 affinity binding sites in SssI (**g**) or TKO DNA (**h**). (**i-j**) Venn diagrams displays the relationship between in vivo mapped Z-DNA sites in activated B-cells and ZBTB43 affinity binding sites in SssI DNA (**i**) or TKO DNA (**j**). Affinity-sequencing results shown represent two biologically independent samples.

**Extended Data Fig. 4:**
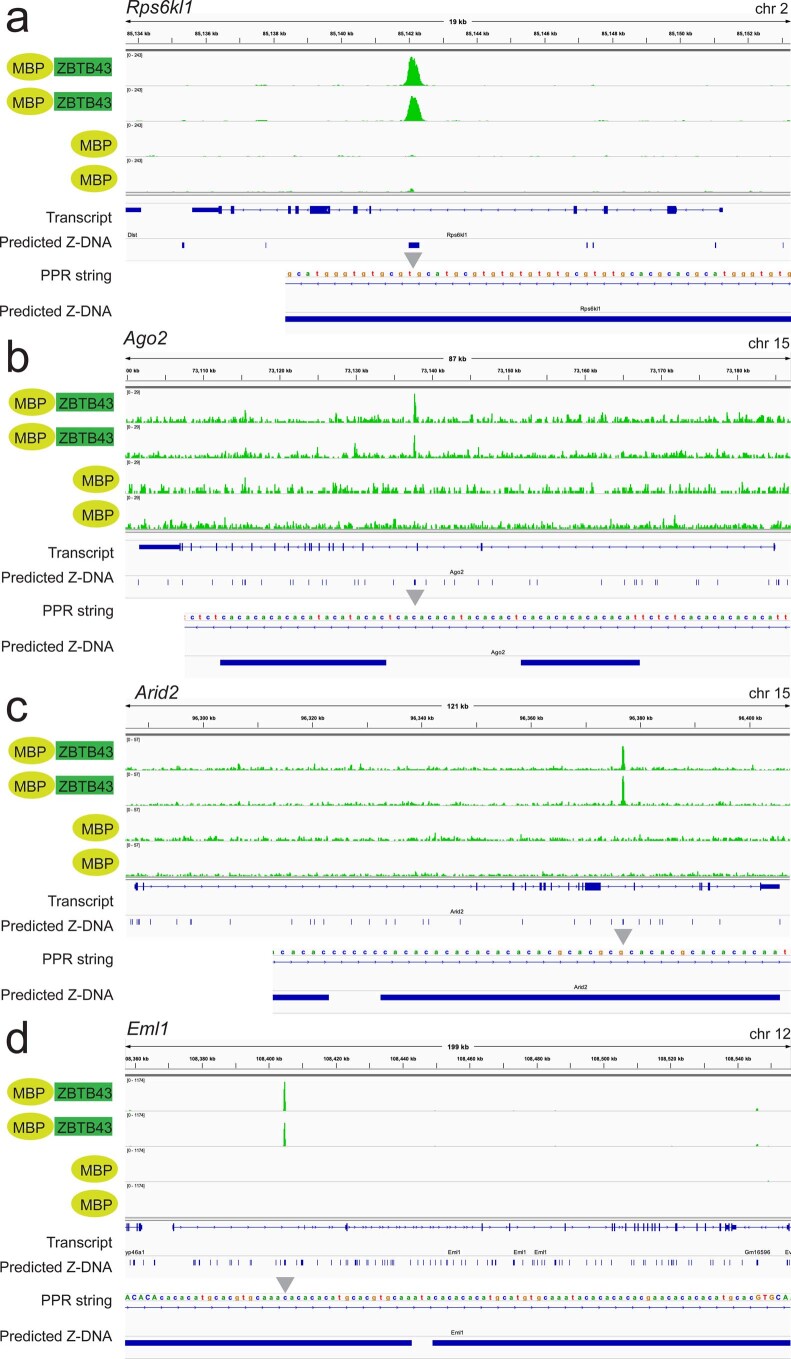
Examples of ZBTB43 affinity binding peaks. IGV browser images of selected specific MBP-ZBTB43 peaks are shown in unmethylated genomic DNA (TKO) at four genomic regions. Control samples show the background of MBP capture. Independent biological replicate samples are displayed. The tracks for transcripts and predicted Z-DNA are displayed at the bottom. The predicted Z-DNA sequence is enlarged to illustrate the underlying PPR sequences. The ZBTB43 peaks are often found in an intron of very long transcripts. (**a**) *Rps6kl1* (**b**) *Ago2* (**c**) *Arid2* (**d**) *Eml1*.

**Extended Data Fig. 5:**
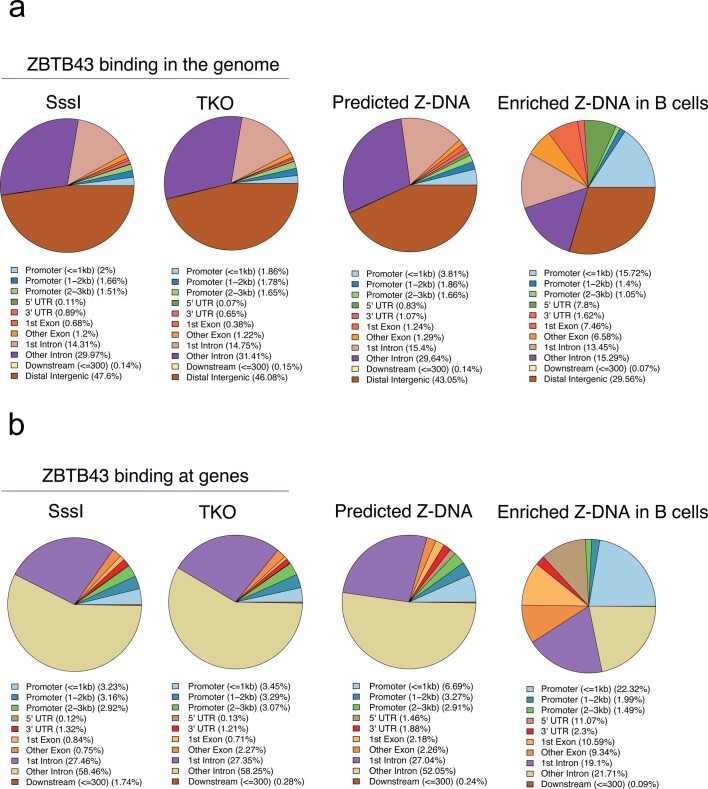
The distribution of ZBTB43 affinity binding peaks closely matches predicted Z-DNA sites. Venn diagrams depict the location of ZBTB43 affinity peaks mapped in fully methylated genomic DNA (SssI) and unmethylated genomic DNA (TKO) relative to the location of transcripts in the genome. In comparison, the distribution of predicted Z-DNA^17^ and enriched Z-DNA mapped *in vivo* in activated B-cells^18^ are also depicted to the right. (**a**) All genomic locations are plotted. (**b**) Distal intergenic regions are excluded. Affinity-sequencing results represent two independent biological replicates.

Owing to its ZFs and nuclear localization, we expected ZBTB43 to bind DNA. We cloned mouse *Zbtb43* complementary DNA by RT–PCR, expressed the MBP-His-tagged ZBTB43 full-length protein (ZBTB43-FL) in *Escherichia coli* and purified it on Ni glutathione agarose beads (Fig. 2a). We also purified the glutathione *S*-transferase (GST)-tagged ZF domain (ZBTB43-ZF) and the maltose-binding protein (MBP)-tagged BTB domain (ZBTB43-BTB) (Fig. 2a). To identify potential binding sites of ZBTB43 in the mouse genome, we carried out in vitro affinity sequencing as detailed in Fig. 2b. We tested binding to fully unmethylated DNA isolated from *Dnmt1*/*Dnmt3a*/*Dnmt3b* triple-knockout (TKO) embryonic stem (ES) cells^16^. We also tested binding to fully methylated DNA prepared with SssI bacterial CpG DNA methyltransferase. We expected that these DNA samples would allow detection of ZBTB43 binding sites and its preference for CpG methylation states in the binding sites. We prepared libraries and deep-sequenced the captured DNA. After aligning the reads to the mouse genome, we identified 14,694 ZBTB43 peaks in the TKO samples (Fig. 2c, Extended Data Fig. 3a and Supplementary Table 1) and 175,775 peaks in the SssI-methylated samples (Extended Data Fig. 3b and Supplementary Table 2). The TKO peaks represented a subset of the SssI peaks (Extended Data Fig. 3c) and the strongest SssI peaks matched with TKO peaks (Extended Data Fig. 3b), suggesting that ZBTB43 binds to unmethylated and methylated DNA, but with higher affinity to methylated DNA. ZBTB43 protein peaks occurred at PPRs such as (CA)_*n*_, (CACG)_4-8_ and (CG)_*n*_ sequences (Fig. 2d). These PPRs are predicted to adopt Z-DNA structures^17^. We examined the ZBTB43 affinity read coverage at the predicted Z-DNA sites in the genome^17^ and at in vivo-mapped (enriched) Z-DNA sites in activated B cells^18^ (Fig. 2e and Extended Data Fig. 3d–j). We found a strong match between ZBTB43 binding and predicted Z-DNA sites (Extended Data Figs. 3–5).

We confirmed the binding of ZBTB43 to selected affinity binding sequences using electrophoretic mobility shift assays (EMSA). The ZBTB43-FL protein or ZBTB43-ZF domain alone specifically shifted fluorescein amidite (FAM)-labelled double-stranded DNA probes (Supplementary Table 3) at the *Rps6kl1* and *Ago2* peaks and the consensus PPRs (CACG)_8_ and (CA)_16_ (Fig. 2g,h). The ZBTB-BTB domain lacked specific DNA binding at the same sequences (Fig. 2h). These experiments collectively demonstrate that ZBTB43 via its ZF domain has affinity to PPRs.

### The ZBTB43 binding sequence is mutagenic

**Fig. 3:**
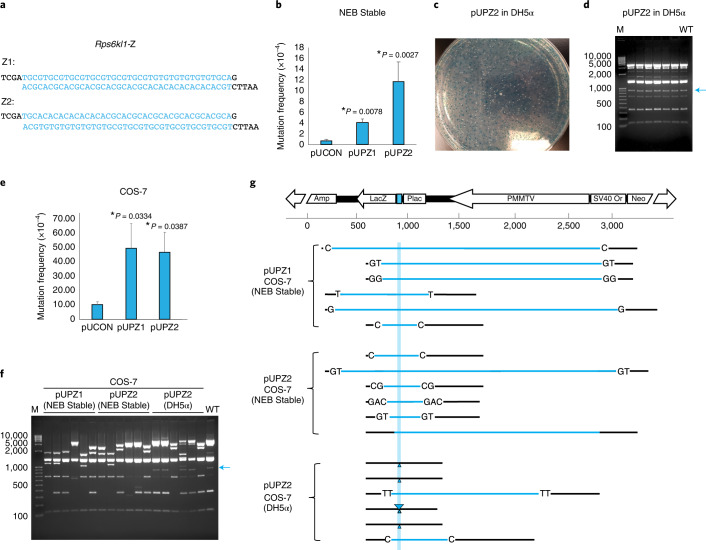
ZBTB43 binding sequences are mutagenic in mammalian cells. **a**, ZBTB43 binding sequence at *Rps6kl1*, in forward and reverse orientation (Z1 and Z2), was inserted into the vector pUCON, and the resulting plasmids pUPZ1 and pUPZ2 were tested in *LacZ* mutation assays. **b**, Mutation frequencies in bacterial NEB Stable cells. Average values of three replicate samples are shown with standard deviation. Statistically significant differences (*P* < 0.05) resulting from *n* = 3 independent experiments are marked. These were obtained using two-tailed Student’s *t*-tests (unequal variance). **c**, The *LacZ* gene is frequently mutated at the Z-DNA insert, as depicted by an agarose plate with DH5α bacteria that contain the parent plasmid (blue colonies) or its mutants (white colonies). **d**, Restriction digestion is shown from randomly picked clones recovered from DH5α bacteria. DNA sequencing revealed small mutations that did not change the size of the 910-bp-long PPR-containing DNA fragment (blue arrow). Molecular size marker is shown in the left lane. Wild-type plasmid (WT) is depicted in the right lane. **e**, Mutation frequencies in mammalian COS-7 cells. Average values of three replicate samples are shown with standard deviation. Statistically significant differences (*P* < 0.05) resulting from *n* = 3 independent experiments are marked. These were obtained using two-tailed Student’s *t*-tests (unequal variance). **f**, Restriction digestion is shown from randomly picked biologically independent clones recovered from COS-7 cells and grown in NEB Stable or DH5α bacteria. The loss of the 910-bp-long PPR-containing fragment (blue arrow) reveals large deletions. Molecular size marker is shown in the left lane. Wild-type plasmid (WT) is depicted in the right lane. Restriction digestion results in **d** and **f** are shown from one of three independent mutagenesis experiments. **g**, Mutation detection in the pUPZ1 and pUPZ2 plasmids mutagenized in COS-7 cells. Structural elements of the vector plasmid are depicted at the top. Position of the inserted Z1 and reciprocal, Z2 sequences is shown in turquoise. Sequencing results are shown from biologically independent clones recovered in NEB Stable or DH5α bacteria, as indicated. Deletions are marked by blue horizontal bars. Micro-homologies that flank these deletions are shown by the DNA sequence. Sequencing results are shown from one of three independent mutagenesis experiments. Source data

Z-DNA-forming PPRs are known to be mutagenic and to cause genomic instability in cultured mammalian cells^6,7^. Thus, we directly tested the mutagenicity of the ZBTB43 binding site, *Rps6kl1*-Z (Fig. 2f and Extended Data Fig. 4a), in bacterial and mammalian cell mutagenesis assays. We cloned the test sequences, a string of PPRs, (CA)_7_(CACG)_6_ and its reverse complement (Fig. 3a) into a mutation-reporter shuttle vector in front of the *LacZ* gene^7^, generating pUPZ2 and pUPZ1, respectively. We first established the background mutation frequency in NEB Stable and DH5α bacteria (Fig. 3b–d). Compared with the control plasmid, we detected an increased mutation frequency in NEB Stable bacteria using both reporter plasmids (Fig. 3b). The Z2 sequence had an unusually high background mutation frequency in DH5α (Fig. 3c). As seen by restriction digestion, the 910-bp-long fragment that carries the test DNA was present in each of six white DH5α colonies, suggesting that the background mutations were point mutations and small indels (Fig. 3d). Similarly, DNA sequencing detected small indels in the white NEB Stable colonies. pUPZ1 mutations in NEB Stable cells comprised the following: deleted TGCG (one clone), gained TGCG (one), deleted (TGCG)_2_ (four) and deleted CG (one). Background mutations of pUPZ2 in NEB Stable cells comprised the following: deleted (CA)_2_ (three), deleted CA (one), deleted CGCA (two), deleted (CGCA)_2_ (one) and deleted ACGC (one).

Mutation reporters were prepared in Stbl3 bacteria and transfected into mammalian COS-7 cells, recovered 48 h later, and digested with *Dpn*I to eliminate the un-replicated plasmids that carry a bacterial methylation signature. Mutations generated in the mammalian cells were first screened in NEB Stable cells. Mutation frequencies with both the Z1 and Z2 inserts were elevated compared with the control B-DNA-containing reporter, pUCON (Fig. 3e). Restriction digestion of six white mutants from pUPZ1 and pUPZ2 screened in NEB Stable cells revealed large deletions, as indicated by the absence of the 910 bp band in all mutants (Fig. 3f). Many mutants also contained deletions in the top 1.9 kb band, adjacent to the insert, suggesting large deletions of over 1 kb. When mutants were screened in DH5α cells, they revealed deletions, re-arrangements and small indels (Fig. 3g), some of which probably originated in the bacteria. DNA sequencing of the reporter plasmids recovered in NEB Stable bacteria, however, confirmed that the mutations that occurred in COS-7 mammalian cells were large deletions (Fig. 3g). In summary, the mutation assays demonstrated that (1) both Z1 and Z2 directions of the prominent ZBTB43 binding peak are mutagenic in both bacterial and mammalian cells; (2) Z2 is extremely unstable in DH5α cells and is moderately mutagenic in NEB Stable cells; (3) Z1 and Z2 cause small indels in bacteria; and (4) both Z1 and Z2 are highly mutagenic and cause large deletions in mammalian cells. The results collectively support the idea that the PPR-rich binding sites of ZBTB43 are mutagenic and could pose risk to genome integrity in mammalian cells.

### ZBTB43 binding sites can form Z-DNA structures in vitro

**Fig. 4:**
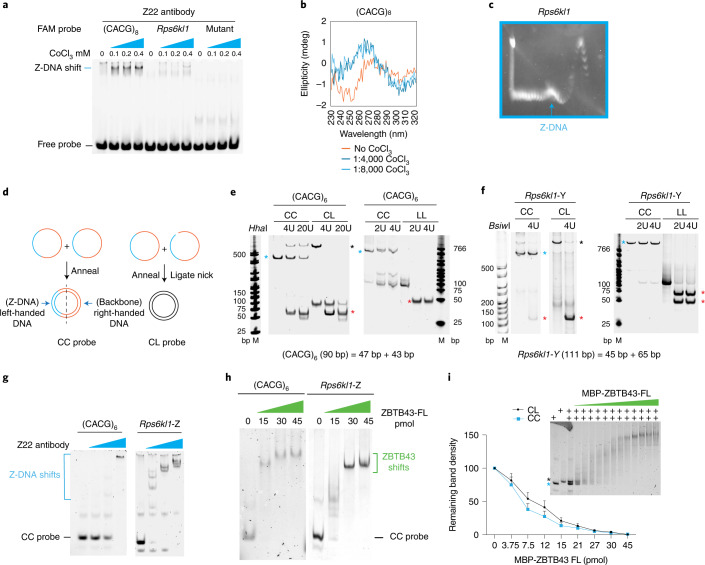
ZBTB43 binding sequences can form the Z-DNA structure in vitro. **a**, EMSA. Hexamine CoCl_3_ was added to the DNA probes at increasing concentrations. The induced Z-DNA was detected as a gel shift using the anti-Z-DNA antibody Z22. **b**, Z-DNA formation (blue lines) is detected by CD spectroscopy at the (CACG)_8_ consensus sequence in response to different concentrations of CoCl_3_. The B-DNA specific spectrum is displayed in red. **c**, 2D gel electrophoresis detects a kink (blue arrow) at certain plasmid topoisomers, a sign of Z-DNA formation. **d**, Generation of circular Z-DNA probe. When two single-stranded circles (CC) are annealed, part of the circle, which contains PPRs is forced into left-handed DNA^21^. Circular B-DNA is prepared by annealing a circular and a linear strand followed by ligating the nick (CL). Linear B-DNA is prepared by annealing two strands of linear DNA (LL)^21^. **e**,**f**, Z-DNA structure is confirmed in the CC probe by its insensitivity to restriction enzymes. The CC, CL and LL probes of PPR sequences, as marked above, were subjected to restriction digestion. The CC form (turquoise asterisk), was refractory. The CL form (black asterisk) is linearized, and the LL form is restricted to two fragments (red asterisks). Experiments where the (CACG)_6_ sequence (**e**), and the *Rps6kl1*-Y peak sequence (**f**) are digested using *Hha*I and *Bsiw*I are displayed. **g**, Z-DNA is formed in the CC probe at the ZBTB43 consensus sequence. Increasing amount of the Z-DNA antibody Z22 quantitatively shifts the CC probe of (CACG)_6_ and *Rps6kl1*-Z sequences. **h**, ZBTB43 binds Z-DNA. EMSA results show that ZBTB43-FL shifts the CC probe containing the (CACG)_6_ and *Rps6kl1*-Z sequences. Data shown represent three independent experiments in **a**–**c** and **e**–**h**. **i**, ZBTB43 binds both Z-DNA and B-DNA but prefers Z-DNA. Competition binding experiment is shown where the CC and CL probes were mixed at equal molar ratios and the aliquots were allowed to interact with increasing amounts of ZBTB43-FL before separating the free probes and complexes in EMSA gels. The remaining free CC and CL probes in each reaction were quantified in the gel images. Data are presented as mean ± standard error of the mean (s.e.m.) from *n* = 3 independent experiments. Source data Source data

PPRs are known to easily adopt and stably maintain Z-DNA structures^19^. It is thought that the mutagenicity of PPRs is due to their capacity to form the Z-DNA structure, which is processed in a mutagenic fashion by DNA repair proteins^6^. To test whether ZBTB43 binding sequences could adopt a Z-DNA structure, we performed a gel-shift experiment in the presence of hexamine cobalt chloride, which is known to induce Z-DNA formation on appropriate sequences in vitro. After adding increasing concentrations of cobalt salt, the anti-Z-DNA antibody Z22 recognized increasing amounts of Z-DNA, seen as slower-migrating bands of both the consensus (CACG)_6_ sequence probe and the *Rps6kl1* probe, but not the mutant probe (Fig. 4a). We confirmed Z-DNA formation of the ZBTB43 recognition sequences using independent approaches, including circular dichroism (CD) spectroscopy, and two-dimensional (2D) gel electrophoresis. Figure 4b shows the shift in the absorption spectrum of the (CACG)_8_ consensus sequence between the B-DNA form and the Z-DNA form after induction by CoCl_3_. The 2D gel in Fig. 4c separates the topoisomers of a plasmid that contains the *Rps6kl1* affinity peak sequence. These were generated by topoisomerase 1 (TOPO1) in the presence of increasing concentrations of the intercalator ethidium bromide^20^. The arrow points to an irregularity, which indicates that the DNA was retarded in the first dimension owing to formation of Z-DNA.

In addition, using a recent method^21^, we generated topologically constrained Z-DNA from ZBTB43 binding sequences under physiological conditions. When two single-stranded DNA circles (CC) are annealed, the simple repeat sequences snap into left-handed Z-DNA in one half of the circle, while the other half of the circle remains right-handed B-DNA (Fig. 4d). As controls for the CC-containing constrained Z-DNA, we generated a flexible B-DNA circle by annealing a circular and a linear single-stranded DNA molecule (CL), and then ligating the second strand (Fig. 4d). We also generated B-DNA by annealing two linear DNA strands (LL). Z-DNA is known to be resistant to restriction enzyme digestion^22,23^. We used restriction digestion to confirm the presence of Z-DNA structure in the CC probes of the (CACG)_6_ and Rps6kl-Y sequences (Fig. 4e,f). Control B-DNA templates of the same sequences were sensitive to restriction digestions (Fig. 4e,f). Using EMSA we found that CC probes containing the ZBTB43 consensus sequence (CACG)_6_ or the *Rps6kl1*-Z affinity peak sequence were fully shifted by the Z-DNA antibody (Fig. 4g); therefore, Z-DNA structure was present in 100% of the CC probes.

We showed earlier that ZBTB43 specifically binds the PPR sequences in linear double-stranded probes (Fig. 2g), which are in B-DNA form. To determine whether ZBTB43 binds PPRs in the Z-DNA structure, we incubated ZBTB43-FL with the CC probes, which contain topologically constrained Z-DNA. We found that the ZBTB43-FL protein recognized and strongly bound the CC probes containing the (CACG)_6_ sequence or the ZBTB43 binding site at *Rps6kl1*-Z (Fig. 4h). These results suggest that ZBTB43 has the capacity to bind PPRs in the Z-DNA conformation.

To assess the relative affinity of ZBTB43 to Z-DNA and B-DNA, we carried out a competition EMSA assay using increasing amounts of ZBTB43-FL with the CC and CL probes combined at 1:1 ratio. The CC probe was bound more readily than the CL probe in three separate experiments (Fig. 4i). These results indicate that ZBTB43 binds PPRs in both B-DNA and Z-DNA structures but prefers Z-DNA over B-DNA.

### ZBTB43 alters DNA topology and removes Z-DNA in vitro

**Fig. 5:**
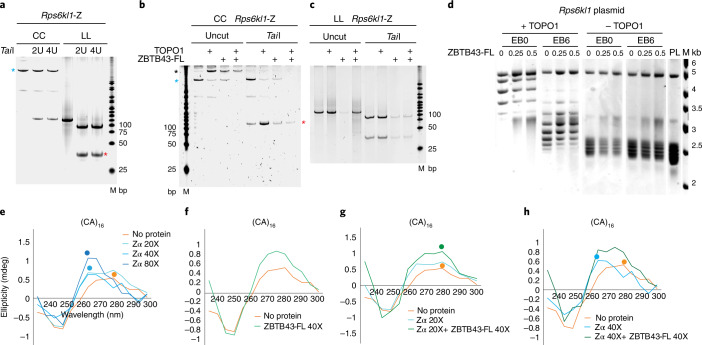
ZBTB43 removes Z-DNA in vitro. **a**–**c** show that ZBTB43 has the capacity to remove Z-DNA in vitro. **a**, Restriction digestion using *Tai*I of the *Rps6kl1*-Z affinity peak in the CC probe (blue asterisk) and the LL probe confirms Z-DNA and B-DNA, respectively. **b**, ZBTB43 renders CC sensitive to digestion. The CC probe was reacted with TOPO1, or ZBTB43, or both, followed by phenol extraction and precipitation. The resulting DNA was run on a gel before or after *Tai*I digestion. TOPO1 and/or ZBTB43 resulted in a band that migrated slower than the CC form (black asterisk). *Tai*I produced a linear 121 bp fragment (red asterisk). **c**, ZBTB43 has no effect on B-DNA. The LL probe (121 bp) was reacted with TOPO1, and/or ZBTB43, and the recovered LL DNA was still sensitive to *Tai*I digestion into two fragments of 37 and 84 bp. **d**, ZBTB43 enhances the effect of TOPO1 in reducing supercoiling-induced tension. TOPO1 was reacted with plasmid DNA containing the *Rps6kl1* affinity peak sequence in the presence or absence of ethidium bromide (EB6 or EB0, respectively) and increasing amounts of ZBTB43-FL. Control reactions were run without TOPO1. Supercoiled plasmid (PL) purified from bacteria and a molecular weight marker (M) are included. **e**–**h** show that ZBTB43 reverses the action of ADAR1 Z-α domain on DNA topology in vitro. **e**, To induce Z-DNA, the Z-α domain was added to the (CA)_16_ linear DNA probe in increasing molar excess. B-DNA specific spectrum, peaking at 280 nm (orange dot) is eliminated by 20× excess of Z-α. New Z-DNA peaks (turquoise and blue dots) at 260 nm are visible at 40× and 80× excess of Z-α. **f**, ZBTB43 has no effect on B-DNA topology. **g**, Forty-fold molar excess of ZBTB43 reverses the Z-to-B shift caused by 20-fold molar excess of Z-α (280 nm peak regained, green dot). **h**, Forty-fold molar excess of ZBTB43 reverses the Z-to-B shift caused by 40-fold molar excess of Z-α (ZBTB43 reverts the peak from 260 nm). Data shown represent two (**a**–**c**) or three (**d**) independent experiments. Experiments in **e**–**h** have been performed once, and each CD spectrum is presented as an average of three scans. Source data

We utilized the known property of Z-DNA structure to resist restriction enzyme digestion^22,23^ to test whether ZBTB43 can alter the Z-DNA structure. We first confirmed the resistance of the mutagenic *Rps6kl1*-Z sequence in the CC but not LL probe to *Tai*I digestion (Fig. 5a). We then subjected the CC or LL probes to reactions with topoisomerase I (TOPO1) or ZBTB43-FL, or both, followed by isolating the resulting DNA and digesting it with *Tai*I restriction enzyme (Fig. 5b,c). In the control experiment, TOPO1 and/or ZBTB43 had no effect on the sensitivity of the LL probe to *Tai*I (Fig. 5c). However, TOPO1 and ZBTB43 reduced the intensity of the CC band, which retained resistance to *Tai*I (Fig. 5b). A slower-migrating band appeared at the same time, which was sensitive to *Tai*I, and therefore was B-DNA. Adding ZBTB43-FL alone also resulted in a slower-migrating, restriction-sensitive band not seen in the control, implying that ZBTB43-FL has the ability to remodel Z-DNA on its own in vitro.

Z-DNA structure formation at PPRs is induced by DNA supercoiling^24^. We hypothesized that ZBTB43 removes Z-DNA structures by affecting DNA topology. TOPO1 facilitates the release of superhelical tension from DNA by generating single-strand nicks, rotating one strand around the other, and re-ligating the nick. When we included ZBTB43 in the reaction of TOPO1 and the plasmid DNA containing the *Rps6kl*1 affinity peak sequence, we found that the range of topoisomers shifted to less superhelical topoisomers (Fig. 5d). Adding ethidium bromide intercalator in this reaction resulted in a different set of topoisomers, as expected. Again, the presence of ZBTB43 resulted in a shifted pattern of topoisomers compared to TOPO1 alone. In this experimental system, ZBTB43 enhanced the relaxing effect of TOPO1 on DNA topology (Fig. 5d).

The Z-α domain of the cytoplasmic ADAR1 protein is known to convert B-DNA to Z-DNA^25^ in vitro. This provided us the opportunity to test the effect of ZBTB43 on Z-DNA using CD. We first confirmed that Z-α shifts the ellipticity of the (CA)_16_ double-stranded linear DNA to the lower wavelength (Fig. 5e), by generating Z-DNA from B-DNA. We also confirmed that ZBTB43 does not shift the ellipticity of the same probe (Fig. 5f), therefore, it has no effect on the B-DNA structure. When we co-incubated Z-α and ZBTB43 with the (CA)_16_ probe, the effect of Z-α was eliminated (Fig. 5g,h), suggesting that ZBTB43 reversed the effect of Z-α on B-DNA. The results above collectively suggest that ZBTB43 is capable of altering Z-DNA structures in vitro and has a similar effect on DNA topology as TOPO1.

### ZBTB43 removes Z-DNA structures in prospermatogonia

**Fig. 6:**
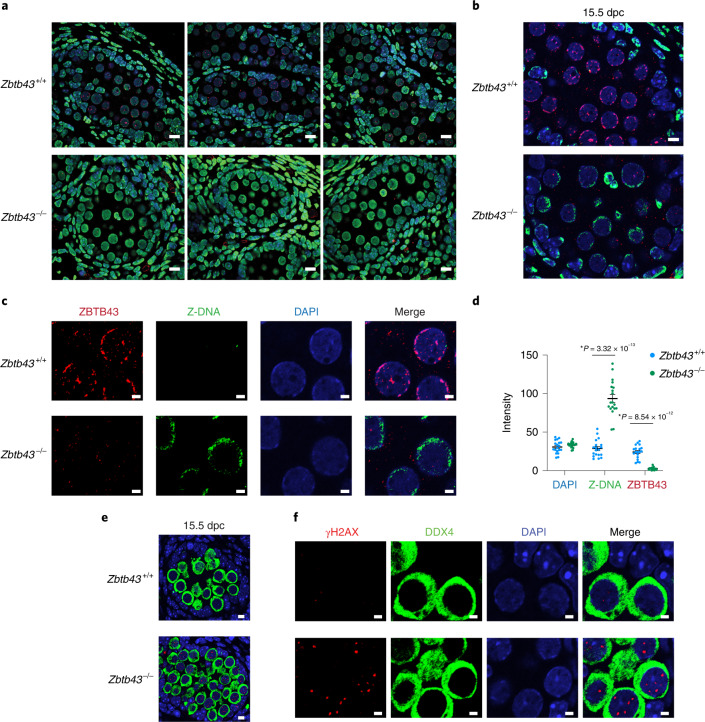
ZBTB43 removes Z-DNA in prospermatogonia and protects from DSBs. **a**, Global Z-DNA is eliminated by ZBTB43 in *Zbtb43*^+/+^, but not in *Zbtb43*^−/−^ foetal testis sections at 15.5 dpc. Images of testicular cords were obtained by immunohistochemistry and confocal microscopy using the ZBTB43 (red), and Z-DNA (green) antibodies, counterstained with DAPI. Scale bars, 10 µm. **b**, Images of testicular cords as above are shown using higher magnification and background reduction. Scale bar, 5 µm. **c**, Images of germ cells as above are shown using higher magnification. Scale bars, 2 µm. **d**, Quantification of the fluorescence intensities of DAPI, Z-DNA and ZBTB43 immunostaining are depicted in 15.5 dpc *Zbtb43*^+/+^ and *Zbtb43*^−/−^ prospermatogonia. Spermatogonia (*n* = 10 + 9) were quantified from two independent foetuses for each genotype. The intensity was measured by Fiji, and the quantification was done by Prism. Data are presented as mean ± s.e.m. The differences between genotypes were statistically significant for the Z-DNA and ZBTB43 intensities by multiple unpaired two-tailed *t*-tests. **e**, ZBTB43 protects from DSBs. Immunohistochemistry of the 15.5 dpc *Zbtb43*^+/+^ and *Zbtb43*^−/−^ foetal testis sections is shown using the γH2AX (red) and DDX4 (green) antibodies, counterstained with DAPI. Scale bars, 5 µm. **f**, Enlarged details of the testicular cords are displayed. Scale bars, 2 µm. The results shown represent three (**a**–**c**) or two (**e**,**f**) independent experiments done using four biologically independent testis samples per experiment. Source data

**Extended Data Fig. 6:**
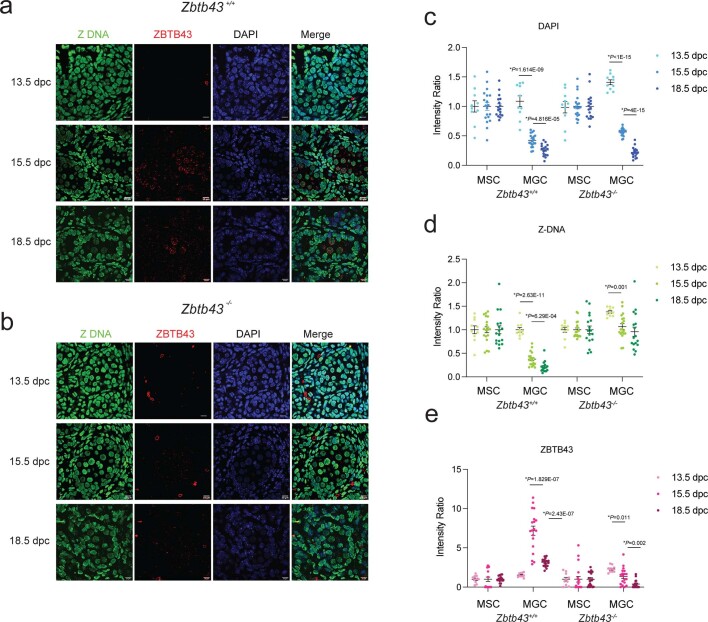
Z-DNA is removed in prospermatogonia between 13.5 and 15.5 dpc. (**a-b**) Immunohistochemistry is shown of the *Zbtb43*^+/+^ wild-type (**a**), and *Zbtb43*^-/-^ mutant (**b**) fetal testis sections at 13.5, 15.5 and 18.5 dpc using the ZBTB43 (red), and Z-DNA (green) antibodies, counterstained with DAPI. Scale bar: 10 µm. The ZBTB43-Z-DNA double staining was done three times at all stages. (**c-e**) Quantification of signal intensities of DAPI, Z-DNA and ZBTB43 in *Zbtb43*^+/+^ wild-type, and *Zbtb43*^-/-^ mutant fetal male germ cells shown relative to the corresponding somatic cells at 13.5 dpc (n=10 cells from one out of two independent experiments) and at 15.5 and 18.5 dpc (n=19 cells from one out of two independent experiments). Data are presented as mean values +/- SEM. Statistically significant changes as calculated by multiple 2-sided T-tests are marked with *P*-value. Source data

To test the role of ZBTB43 on remodelling Z-DNA in prospermatogonia in vivo, we immunostained paraffin sections of wild-type and *Zbtb43*^−/−^ foetal mouse testes at 15.5 dpc with antibodies against ZBTB43 and Z-DNA (Fig. 6a–d). The ZBTB43-negative mutant germ cells exhibited a strikingly higher level of Z-DNA staining compared with ZBTB43-positive wild-type germ cells (Fig. 6d). We similarly stained foetal testis sections at 13.5, 15.5 and 18.5 dpc and found that the mutant germ cells were affected at 15.5 dpc (Extended Data Fig. 6), the time at which ZBTB43 expression is turned on.

### ZBTB43 protects foetal MGCs from DNA breaks

Z-DNA-forming PPRs are known to stimulate the formation of DSBs. We found that the sequence at a prominent ZBTB43 site was highly mutagenic in mammalian cells (Fig. 3). We next tested whether an increased Z-DNA load in the absence of ZBTB43 renders the *Zbtb43*^−/−^ mutant foetal MGCs more vulnerable to DNA breakage. We performed immunostaining using an antibody against γH2AX, a histone variant that is a marker for DSBs. Formation of γH2AX foci increased in *Zbtb43*^−/−^ mutant germ cells compared with wild-type germ cells (Fig. 6e,f), suggesting that ZBTB43 protein protects the genome from DSBs in foetal MGCs in vivo.

### ZBTB43 binds at PPRs in prospermatogonia

**Fig. 7:**
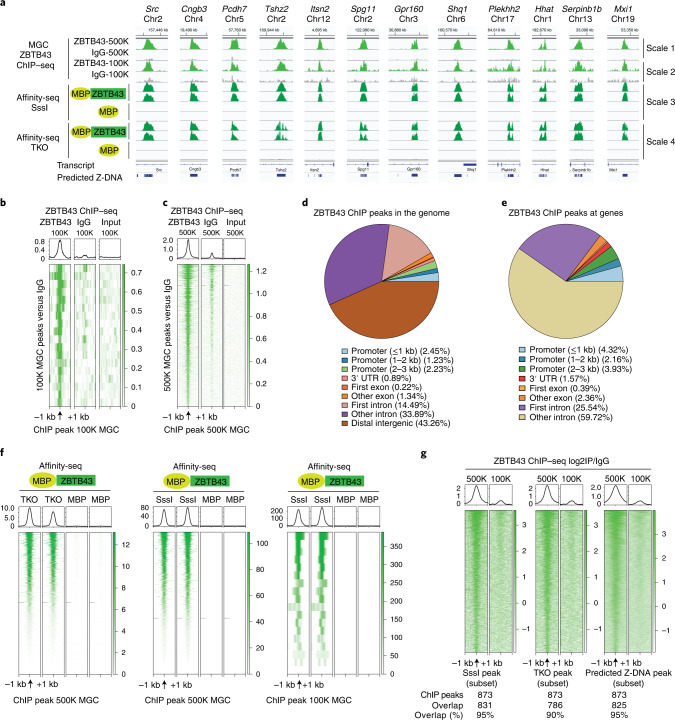
ZBTB43 binds at PPRs in prospermatogonia in vivo. **a**, Genome browser images of ZBTB43 ChIP–seq peaks (light green) obtained in purified 15.5 dpc prospermatogonia are displayed at 12 genomic locations. IgG lanes are shown as controls. The ChIP peaks align with the in vitro affinity sequencing peaks found in fully methylated genomic DNA (SssI) and in fully unmethylated genomic DNA (TKO) (dark green). MBP affinity-seq lanes are shown as controls. The scale of reads was normalized between experimental samples and their respective background control samples using the ‘group-autoscale’ function of IGV as marked on the right. **b**,**c**, Heatmap showing the ZBTB43 ChIP–seq peaks detected against IgG background in 100,000 (**b**) or 500,000 (**c**) purified prospermatogonia. The read intensities in three libraries, ZBTB43 antibody, IgG antibody and input DNA (as marked above), are plotted centred at the peak and using +1 kb and −1 kb flanking regions. **d**,**e**, Venn diagrams depicting the location of ZBTB43 ChIP–seq peaks mapped in 15.5 dpc prospermatogonia relative to the location of transcripts in the genome. In **d**, all genomic locations are plotted. In **e**, distal intergenic regions are excluded. **f**, The ZBTB43 ChIP–seq peaks detected in vivo are recognized by purified ZBTB43 protein in vitro. Heatmaps display the read intensities of ZBTB43-FL and the control MBP in affinity binding with unmethylated DNA (TKO) or methylated DNA (SssI). The plotted regions were centred at ChIP peaks called in 500,000 or 100,000 prospermatogonia against IgG, as indicated at the bottom. **g**, The ZBTB43 ChIP–seq peaks detected in prospermatogonia align with affinity-seq peaks and with predicted Z-DNA. Heat maps display the ChIP–seq log_2_ IP/IgG read intensities detected in 500 K or 100 K prospermatogonia (as marked at the top) along subset of genomic regions centred at ZBTB43-FL affinity-seq peaks in methylated DNA (SssI), unmethylated DNA (TKO) and at the subset of predicted Z-DNA sites (marked at the bottom) where overlap is found with ChIP–seq peaks. The ChIP–seq and affinity-sequencing results shown represent two independent biological replicates in **a**, **f** and **g**.

To detect in vivo binding sites of ZBTB43, we isolated prospermatogonia at 15.5 dpc by fluorescence-activated cell sorting (FACS), based on the Oct4-EGFP transgene expression^15^, and performed chromatin immunoprecipitation followed by sequencing (ChIP–seq) experiments as we did earlier^26^ using the anti-ZBTB43 antibody. We found that specific regions, such as *Src*, *Cngb3*, *Pcdh7* and *Tshz2*, displayed clear in vivo ChIP–seq peaks in wild-type prospermatogonia (Fig. 7a). We identified 897 peaks against a non-specific rabbit IgG ChIP–seq library (Supplementary Table 4), and 878 of these matched the input-controlled peaks (*n* = 1,183). The peaks called using 100,000 prospermatogonia (Fig. 7b) represent a subset of peaks called in 500,000 prospermatogonia (Fig. 7c). The distribution of ChIP peaks in the genome (Fig. 7d) and at genes (Fig. 7e) closely matches that of the affinity peaks and predicted Z-DNA sites (Extended Data Fig. 5). The ChIP–seq peaks overlap with a subset of ZBTB43 affinity peaks (Fig. 7a,f,g) and predicted Z-DNA sites (Fig. 7g). The ChIP–seq data show that ZBTB43 specifically binds PPRs in 15.5 dpc prospermatogonia in vivo.

### ZBTB43 is required for de novo DNA methylation at PPRs

**Fig. 8:**
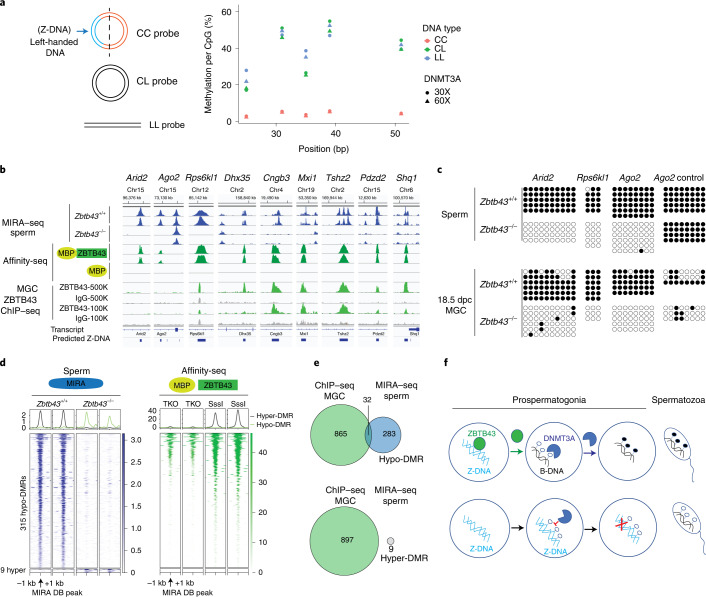
ZBTB43 is required for de novo methylation of PPRs in prospermatogonia. **a**, Z-DNA structure inhibits DNMT3A catalytic activity in vitro. The CC, CL and LL substrates were prepared from the *Rps6kl1* affinity binding sequence and were subjected to methylation by DNMT3A at 30× or 60× molar excess. The level of DNA methylation at each CpG is plotted after multiplexed bisulfite sequencing in two biologically independent replicates. **b**, DNA methylation is aberrant in sperm of *Zbtb43*^−/−^ mice at the sites of ZBTB43 binding. IGV browser images display selected in vivo hypomethylated regions detected using MIRA–seq assays in *Zbtb43*^−/−^ versus wild-type spermatozoa (navy). The ZBTB43 affinity binding peaks (green), and ChIP–seq peaks mapped in 15.5 dpc prospermatogonia (light green) are also shown together with the MBP and IgG backgrounds. Independent replicate samples are displayed. **c**, DMRs originate at the time of de novo methylation in prospermatogonia. Bisulfite sequencing results show methylated and unmethylated CpGs (black and white circle, respectively) along individual chromosomes at specific DMR sequences and at one control region in adult spermatozoa (top) and 18.5 dpc prospermatogonia (bottom). The genotypes are marked on the left. **d**, Heatmap displaying the MIRA–seq intensities in navy at the hypomethylated and hypermethylated DMRs identified between *Zbtb43*^−/−^ and *Zbtb43*^+/+^ spermatozoa. Heatmaps to the right in green show the affinity binding of MBP-ZBTB43-FL in unmethylated and methylated genomic DNA (TKO and SssI) centred at the sites of sperm DMRs. The sequencing results shown represent two independent biological replicates in **b** and **d**. **e**, ZBTB43 ChIP–seq peaks in 15.5 dpc prospermatogonia overlap with hypo-DMRs detected in *Zbtb43*^−/−^ sperm DNA. Venn diagrams. **f**, Model. Top: ZBTB43 binds Z-DNA in normal prospermatogonia. By removing Z-DNA, ZBTB43 creates an accessible substrate for DNMT3A and facilitates DNA methylation establishment at PPR-rich DNA regions. Bottom: Z-DNA is not removed in the *Zbtb43*^−/−^ prospermatogonia in the absence of ZBTB43, DNA methylation is not established at PPRs, and hypomethylated DMRs are found in mutant sperm. One prominent ZBTB43 region can induce large re-arrangements in mammalian mutation assays, and ZBTB43 protects from DSBs by directly binding to PPRs and removing mutagenic Z-DNA structures in the foetal germ cells.

ZBTB43 in vivo binding at PPRs (Fig. 7) may result in the global removal of Z-DNA in wild-type prospermatogonia as seen by immunohistochemistry (Fig. 6). To detect specific sequences where ZBTB43 has in vivo activity in the germ line, we examined the effect of ZBTB43 on DNA methylation. Earlier studies showed that Z-DNA inhibits the catalytic activity of the DNA methyltransferase enzyme DNMT1 (ref. ^27^). We hypothesized that Z-DNA may also inhibit the catalytic activity of DNMT3A, the DNA methyltransferase enzyme that carries out de novo DNA methylation in prospermatogonia^28^. We first tested whether Z-DNA prevents DNA methylation by DNMT3A. We prepared three different substrates from the *Rps6kl1* affinity-peak region that contains the (CA)_6_(CACG)_6_ repeat sequences: constrained circular Z-DNA (CC), control circular B-DNA (CL) and linear DNA (LL) according to a published approach^21^ (Figs. 4d and 8a). We subjected these substrates to methylation by DNMT3A and found that the Z-DNA-containing CC, but not the CL or LL substrate, was refractory to DNMT3A in vitro (Fig. 8a).

We further hypothesized that Z-DNA affects de novo methylation by DNMT3A in vivo, and ZBTB43 is required in the male germ line for methylating sequences that adopt Z-DNA structure. We thus collected spermatozoa from *Zbtb43*^−/−^ and control *Zbtb43*^+/+^ mice and captured and deep-sequenced the methylated DNA fraction in a MIRA–seq experiment^29^. We detected specific DMRs that lacked DNA methylation in the *Zbtb43*^−/−^ sperm at prominent ZBTB43 affinity binding peaks, such as the introns of *Arid2*, *Ago2* and *Rps6kl1* (Fig. 8b) and confirmed those by bisulfite sequencing (Fig. 8c).

We showed above that Z-DNA removal takes place in prospermatogonia at 15.5 dpc (Fig. 1a), which coincides with the time of global de novo DNA methylation in those cells between 15.5 and 18.5 dpc (refs. ^13,14^). To test whether the sperm DMRs originate at the foetal germ cell stage and if ZBTB43 affects the process of de novo methylation, we isolated 18.5 dpc prospermatogonia from *Zbtb43*^−/−^ and control *Zbtb43*^+/+^ male foetuses by FACS and carried out bisulfite sequencing at selected hypomethylated DMR sequences. In the absence of ZBTB43, the mutant foetal germ cells failed to achieve de novo DNA methylation at the *Arid2*, *Ago2* and *Rps6kl1* MIRA DMRs (Fig. 8c).

**Extended Data Fig. 7:**
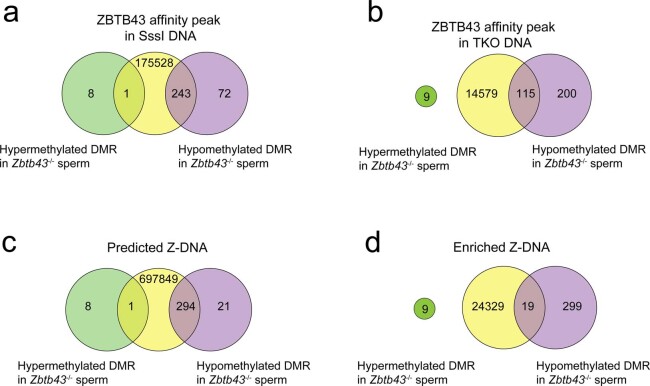
ZBTB43 has affinity to hypomethylated DMRs found in *Zbtb43*^-/-^ sperm, which are rich in Z-DNA sites. (**a**) Venn diagram displays the relationship between ZBTB43 affinity peaks in fully methylated (SssI) genomic DNA and DMRs detected in *Zbtb43*^-/-^ sperm. (**b**) Venn diagram displays the relationship between ZBTB43 affinity peaks in fully unmethylated (TKO) genomic DNA and DMRs detected in *Zbtb43*^-/-^ sperm. (**c**) Venn diagram shows the relationship between predicted Z-DNA sites in the genome and DMRs detected in *Zbtb43*^-/-^ sperm. (**d**) Venn diagram shows the relationship between enriched Z-DNA sites in activated B-cells in the genome^18^ and DMRs detected in *Zbtb43*^-/-^ sperm.

The hypomethylated DMRs (Supplementary Table 5) globally aligned with ZBTB43 in vitro affinity peaks (Fig. 8d and Extended Data Fig. 7a,b), matched with a subset of ChIP–seq peaks (Fig. 8e) and mapped at predicted Z-DNA sites (Extended Data Fig. 7c,d). Examples of hypomethylated regions that overlapped with ChIP–seq peaks are displayed at *Dhx35*, *Cngb3*, *Mxi1*, *Tshz2* and *Pdzd2*, and downstream of *Shq1* (Fig. 8b). Overlap of ZBTB43 ChIP–seq and MIRA hypomethylation DMRs gives direct evidence that methylation of PPRs depends on ZBTB43 binding at those sequences in the male germ line in vivo. Overlap of ZBTB43 affinity-seq peaks and hypomethylated DMRs gives indirect but in vivo evidence that methylation of CpG-containing PPR at a larger number of DMRs depends on ZBTB43 in the male germ line.

### Importance of ZBTB43 in mouse viability and growth

**Extended Data Fig. 8:**
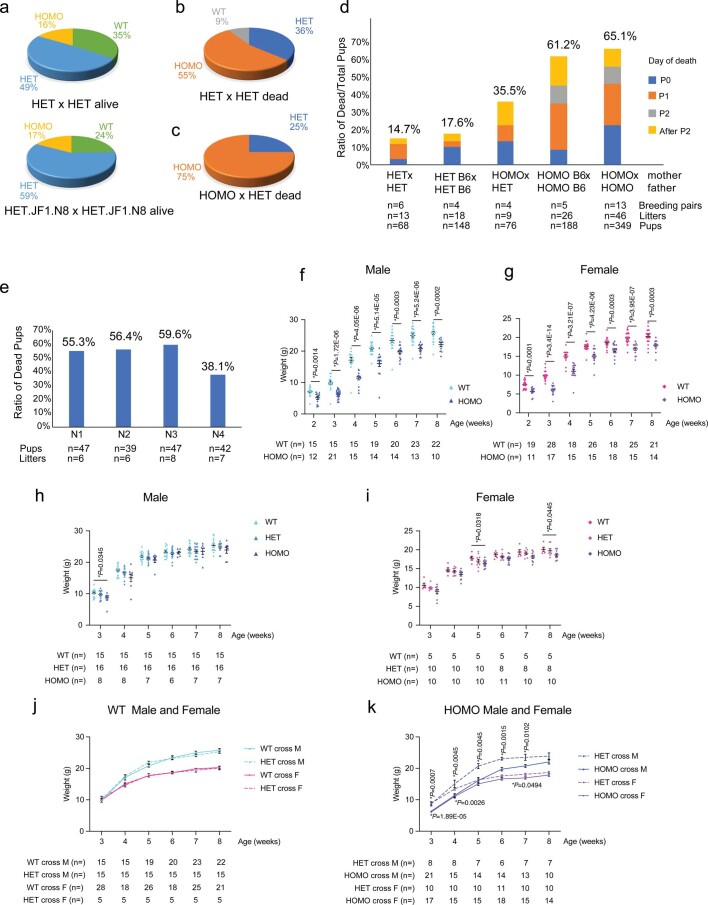
Growth and lethality phenotypes associated with the *Zbtb43* mutation in the soma and germ line. (**a**) *Zbtb43*^-/-^ pups out of the test cross are underrepresented at weaning. Venn diagram shows the distribution of genotypes at weaning from 55 pups out of 6 breeding pairs and 13 litters of the original *Zbtb43*^+/-^ heterozygous stock (HET x HET). The genotype distribution of live weanlings is also plotted from a test cross where the parents had been back-crossed 8-times in the JF1/Ms mouse strain (HET.JF1.N8 x HET.JF1.N8). These results were obtained from four breeding pairs, 19 litters and 70 pups. (**b**) *Zbtb43*^-/-^ pups are overrepresented among dead pups out of the HET x HET cross. Venn diagram shows the distribution of dead pups by genotype. We identified 6 *Zbtb43*^-/-^ dead pups out of 11 total dead pups from 2 litters of 1 breeding pair of the HET x HET initial cross by PCR genotyping. After backcrossing to C57BL/6N once, we identified 10 *Zbtb43*^-/-^ dead pups out 23 dead pups from 20 litters of 4 pairs of HET.B6 X Het B6. (**c**) *Zbtb43*^-/-^ dead pups (n=16) are overrepresented among all dead pups out of the HOMO x HET cross. Venn diagram shows the distribution of dead pups by genotype. (**d**) Partially penetrant lethality in the *Zbtb43* mutant mouse line depends on parental genotype. The portion of newborn pups that died is depicted by bar graphs. The lethality phenotype is shown according to different parental crosses as indicated below each bar. The postnatal day of death (P0, P1, P2 and after P2) is shown by colored columns as coded to the right. *Zbtb43*^-/-^ (HOMO), and *Zbtb43*^+/-^ (HET) mice were obtained from the original JAX stock. Another set of mice were obtained after crossing to C57Bl/6N (B6) one time (HOMO B6, and HET B6). The mother is written first in each cross. The number of breeding pairs, litters, and pups born are provided under the chart. (**e**) The partial perinatal lethality phenotype of *Zbtb43*^-/-^ pups persists through generations (N1, N2, N3 and N4) in the HOMO x HOMO crosses. Total number of animals and litters in each generation are marked under the plot. (**f-g**) *Zbtb43*^-/-^ pups from *Zbtb43*^-/-^ parents exhibit reduced growth compared to *Zbtb43*^+/+^ pups out of *Zbtb43*^+/+^ parents. Weight of male (**f**) and female (**g**) pups is plotted at weeks 2, 3, 4, 5, 5, 6, and 8 after birth, and numbers of pups included in the measurements are given under the dot plots. Data are presented as mean values +/- SEM. Statistical analysis between genotypes at each age was done using multiple two-sided T-Tests. (**h-i**) *Zbtb43*^-/-^ pups exhibit reduced growth after weaning compared to their littermates. Growth of male (**h**) and female (**i**) pups out of HET x HET cross is depicted. Weight of WT, HET and HOMO pups is shown as coded by colors to the right and numbers of pups are given under the dot plots. Data are presented as mean values +/- SEM. Statistically significant difference is marked with *P*-value as determined using multiple two-sided T-Tests between pairs of genotypes at each age. (**j**) Parental genotype of *Zbtb43*^+/+^ pups does not affect their growth. Growth curve of male and female WT pups is shown from 3 weeks to 8 weeks. Solid lines indicate the average weight of WT pups out of WT parents. Dashed lines indicate the average weight of WT pups out of the HET x HET cross. Data are presented as mean values +/- SEM. No statistically significant difference was found between crosses using multiple 2-sided T-Tests. (**k**) Parental genotype of male and female *Zbtb43*^-/-^ pups affects their growth after weaning. Growth curve of male and female HOMO pups is shown from 3 weeks to 8 weeks. Solid lines indicate the average weight of HOMO pups out of HOMO x HOMO cross. Dashed lines indicate the average weight of HOMO pups out of the HET x HET cross. Data are presented as mean values +/- SEM. Statistically significant differences between crosses at each age as obtained from multiple two-sided T-Tests are marked. Source data

The phenotype of the *Zbtb43* mutant mouse has not been described, and the effect of its mutation on development is unknown. *Zbtb43*^−/−^ pups were underrepresented at weaning in test crosses between *Zbtb43*^+/−^ mice, in two different genetic backgrounds, C57BL/6 and JF1/Ms^30^ (Extended Data Fig. 8a). Conversely, *Zbtb43*^−/−^ pups were overrepresented among dead pups from a test cross between *Zbtb43*^+/−^ × *Zbtb43*^+/−^ mice and also in a test cross between *Zbtb43*^−/−^ × *Zbtb43*^+/−^ mice (Extended Data Fig. 8b,c). These findings were consistent with a partially penetrant perinatal lethality of *Zbtb43*^−/−^ pups. To further assess the partially penetrant lethality phenotype, we set up genetic crosses in different combinations of *Zbtb43*^+/−^ and *Zbtb43*^−/−^ parents from the original line (HET, and HOMO, respectively) and also from a line that was backcrossed to the C57BL/6J (B6) wild-type strain one time (HET B6 and HOMO B6). Having a *Zbtb43*^−/−^ mother in the HOMO × HET cross resulted in 35% lethality of HOMO pups (Extended Data Fig. 8d), but having both a *Zbtb43*^−/−^ mother and *Zbtb43*^−/−^ father in the HOMO × HOMO and HOMO B6 × HOMO B6 crosses increased the postnatal lethality of HOMO pups to above 60% (Extended Data Fig. 8d). This suggests that ZBTB43 has parental effects on viability from both female and male germ lines. The lethality phenotype of HOMO offspring out of the HOMO × HOMO cross was observed in consecutive generations (Extended Data Fig. 8e).

Next, we measured the weight of live offspring of different genotypes weekly between 3 and 8 weeks of age. We found that the weight of HOMO mice out of the HOMO × HOMO cross was significantly lower at each timepoint compared with control WT mice out of WT parents (Extended Data Fig. 8f,g). HET and HOMO mice out of the HET × HET cross also appeared smaller compared with the WT siblings (Extended Data Fig. 8h,i). The difference was significant for HOMO males at 3 weeks and for HOMO females at 5 and 8 weeks. Whereas growth of WT pups was indistinguishable from WT × WT or HET × HET parents (Extended Data Fig. 8j), HOMO pups were smaller from HOMO × HOMO cross than HOMO pups from HET × HET cross (Extended Data Fig. 8k). This suggests that having two *Zbtb43*^−/−^ parents enhances the growth retardation phenotype in pups of the *Zbtb43*^−/−^ genotype. These results are consistent with parental effects of ZBTB43 in the mouse germ line affecting viability and post-weaning growth of the offspring.

## Discussion

The mammalian germ line undergoes global epigenome remodelling at the level of DNA methylation and chromatin structure^11–14^. In the present work, we discovered an additional layer of the remodelling process in the male germ line at the level of DNA structure. We found that the left handed transient Z-DNA structure that forms under permissive conditions^31^ is removed naturally in prospermatogonia between 13.5 dpc and 15.5 dpc (Fig. 1a–d). We also provide genetic, epigenomic and biochemical evidence that a sequence-specific DNA binding protein, ZBTB43, carries out this function, as summarized in the model (Fig. 8f). Remodelling of DNA structure not only coincides with the major wave of global epigenetic remodelling in the foetal male germ line^12–14^, but de novo methylation of PPR-rich regions in prospermatogonia also requires ZBTB43 (Fig. 8b–e). The MIRA–seq experiment identifies ZBTB43’s function for removing Z-DNA structure in the male germ line in vivo at more than 300 sperm DMRs. As methyl-CpG binding proteins used in the MIRA assay prefer higher density of 5mCpGs^32^, our list of DMRs is an underestimation of ZBTB43’s effect on de novo methylation.

Mouse breeding experiments (Extended Data Fig. 8) suggest that ZBTB43 function is important for growth and viability of the offspring in a stochastic manner. Future experiments will address the exact cause of these parental effect phenotypes. It will be interesting to find out whether methylating PPRs itself is important for germline function or DNA methylation may only be a consequence of Z-DNA structure remodelling, and whether ZBTB43 selects those Z-DNA sequences that have evolved to be of value for regulatory purposes, by facilitating essential methylation in this case.

ZBTB43 indirectly affects the function of DNMT3A, the methyltransferase responsible for de novo methylation in prospermatogonia^28,33^. Our biochemical experiments showed that, similar to DNMT1 and bacterial methyltransferases^22,23,27^, DNMT3A discriminates against Z-DNA substrates (Fig. 8a). By removing Z-DNA structures, ZBTB43 generates the favoured B-DNA substrate for DNMT3A, facilitating DNA methylation establishment at specific PPR-rich DNA regions (Fig. 8). This study identifies ZBTB43 as a unique DNA-binding factor required for specifying locations of de novo DNA methyltransferase activity in the male germ line.

Mechanistically, ZBTB43 can reduce the superhelicity of PPR-containing plasmid DNA together with TOPO1 (Fig. 5d). This should destabilize Z-DNA structures, because negative superhelicity stabilizes Z-DNA structure in plasmid DNA in vitro^24,34^ and reducing negative superhelicity in DNA is known to destabilize Z-DNA structure in vivo in bacteria and mammalian cells^35,36^. In addition, ZBTB43 has an ability to eliminate the Z-DNA structure by itself from a topologically constrained circular DNA (Fig. 5b), and it can also reverse the action of Z-α domain of ADAR1 in vitro (Fig. 5e–h). Future biochemical studies will identify interacting molecules and structural biology studies will elucidate the detailed mechanism of how ZBTB43 remodels Z-DNA.

Unlike known Z-DNA binding proteins, such as ADAR1 and E3L, which carry out their biological function in the cytoplasm^37,38^, ZBTB43 is a nuclear protein (Fig. 1d–f) and functions to remove Z-DNA in the nucleus (Fig. 6a–d). Another transcription factor that regulates DNA structure, ZSCAN4, retains (CA)_*n*_ microsatellite repeats in the B-DNA structure by enforcing the nucleosome occupancy at those sequences in ES cells^39^. As ZSCAN4 does not bind the Z-DNA structure, ZBTB43 is the only protein factor, to the best of our knowledge, that binds and eliminates Z-DNA structures in the nucleus.

Z-DNA-forming PPRs are mutagenic and can lead to large deletions and re-arrangements in the mammalian genome^7^. It has been shown that PPRs induce DSBs in mammalian cells, most likely due to their capacity to form Z-DNA^6,7,40^. Indeed, we found that the level of DSBs is elevated in *Zbtb43*^−/−^ mutant prospermatogonia (Fig. 6e,f), where removal of Z-DNA structures does not occur (Fig. 6a–d). This provides genetic evidence for the model that Z-DNA structure, not simply the PPR sequence, poses a risk for DSB formation in the mammalian organism in vivo.

Indeed, the prominent PPR-rich ZBTB43-binding peak from an intron of *Rps6kl1* can induce large re-arrangements in mammalian mutation assays (Fig. 3e–g). It forms Z-DNA in vitro (Fig. 4a–c,g), and it is preferentially bound by ZBTB43 in the Z-DNA structure in vitro (Fig. 4h,i). This sequence also changes its DNA structure from Z to B in response to ZBTB43 in vitro (Fig. 5b). ZBTB43 binds this DNA fragment and changes its conformation in prospermatogonia in vivo, as judged by its effect on de novo DNA methylation (Fig. 8b,c). By removing the mutagenic Z-DNA structure at such sites, ZBTB43 may balance the mutagenicity of PPRs in the germ line, thus controlling the level of mutations passed to the next generation. Therefore, by eliminating the Z-DNA structures in the germ line, ZBTB43 safeguards genome stability of the species.

An interesting aspect is the timing of Z-DNA removal during male gametogenesis. As these MGCs are in mitotic arrest at 15.5 dpc and remain there until after birth, any newly formed DSBs at Z-DNA-forming sequences could only be repaired by the error-prone non-homologous end joining pathway but not by error-free homologous recombination^41,42^. For these reasons, it seems logical that a mechanism for Z-DNA removal during this critical time period has evolved. Future experiments will test whether the DSB formation in the *Zbtb43* mutant foetal germ cells leads to mutations, which then could be passed to the next generation via the sperm.

Repeated loss of pelvic structures in evolution was reported in the stickleback fish, and this corresponded to genomic deletions which occurred at PPRs^40^. In this instance, deletions that occurred at PPRs lent the freshwater fish an advantage, which was selected for during parallel evolution. Such evolutionary events may be controlled differently in different species. It is interesting to note that stickleback fish do not have the *Zbtb43* gene, and without ZBTB43 they may not have a mechanism to curb Z-DNA formation and the mutagenicity of PPRs in the germ line.

In summary, our results reveal the existence of a ZBTB43-dependent mechanism that protects genomic and epigenomic integrity of the species by regulating mutagenic Z-DNA structures at PPRs in the mammalian germ line.

## Methods

### Mice

This research complies with all relevant ethical regulations. All animal experiments were performed according to the National Institutes of Health Guide for the Care and Use of Laboratory animals, with Institutional Care and Use Committee-approved protocols at Van Andel Institute.

#### Mouse lines

The *Zbtb43*^+/−^ heterozygous mouse C57BL/6N-Zbtb43^tm1b(KOMP)Mbp/J^ was obtained from the Jackson Laboratory, and kept in the C57BL/6N genetic background. A subline was created by back-crossing to JF1/Ms^30^ mice. To obtain wild-type prospermatogonia for ChIP–seq by FACS, C57BL/6J females (~2 months old) were crossed with B6;CBA-Tg^(Pou5f1-EGFP)2Mnn^ (TgOG2) (ref. ^15^) transgenic males (2–12 months old) and male foetuses were collected at 15.5 dpc. *Zbtb43*^+/–^; TgOG2 females (~2 months old) were crossed with *Zbtb43*^+/–^; TgOG2 transgenic males (2–12 months old) to obtain mutant and wild-type foetuses at 18.5 dpc, for collecting prospermatogonia by FACS. Adult *Zbtb43*^–/–^ and wild-type males at 3 months of age were used for sperm collection. Mice were kept on a 12-h light/dark cycle, 7:00 to 18:00 at ambient room temperature of 72 degrees F, and at humidity of 30–70%, monitored through the building control management system.

#### Genotyping of *Zbtb43* mutant mice

Mouse tail tip samples were collected at weaning time, boiled in 180 µl tail digestion buffer (50 mM NaOH, 0.1 mM EDTA) at 95 °C for 20 min, and dissociated by vortexing. Then, 20 µl tail neutralization buffer (1 M Tris–HCl pH 8.0) was added to stop the digestion. Two pairs of primers, one for wild type and one for mutant, were mixed with 1 µl digested tail complex in 10 µl total volume including 2× GoTaq Green Master Mix (M712B-C, Promega) using the following PCR cycles: 95 °C, 2 min, (95 °C, 30 s, 58 °C, 30 s, 72 °C, 45 s) × 39, and 72 °C, 5 min. The size of wild-type and mutant bands, 298 bp and 405 bp, respectively, was resolved in a 1.5% agarose gel. The primer sequences are listed in Supplementary Table 3. Genotyping experiments (Extended Data Fig. 2b) were consistently done multiple times.

### Western blot

#### Sample preparation

Brain and kidney were dissected from adult mice. After a 1× PBS rinse, the organ was immediately lysed in RIPA buffer (1 ml for one kidney, 2 ml for one brain) containing protease inhibitor cocktail (Roche, cat. no. 11697498001) and sonicated for 10 s (at an amplitude of 25%) at 4 °C in an EpiShear Probe Sonicator. Sample was centrifuged at 16,800*g* (13,200 rpm) for 20 min at 4 °C in an Eppendorf centrifuge. Protein concentration was determined from the supernatant using Bradford colorimetry. Then, 50 µg total protein per each lane was resolved in a 10% SDS–PAGE and transferred onto a PVDF membrane.

#### Antibody incubation

After blocking with 5% non-fat dry milk in TBST, protein blots were incubated with primary antibody overnight at 4 °C. Then blots were washed three times and probed with secondary peroxidase-conjugated antibodies for 1 h at room temperature. Signals were visualized using an ECL chromogenic kit. Antibodies were diluted in 5% non-fat dry milk in 1× PBS as follows: anti-ZBTB43 (1:100, Aviva Systems Biology, cat. no. ARP39048-P050); anti-LacZ (1: 2,000, Abcam, cat. no. ab9361); anti-GAPDH (1:10,000, Abclonal, cat. no. AC035); and secondary antibodies HRP goat anti-rabbit IgG (1:5,000, Active Motif, cat. no. 15015); and Alexa Fluor 488 goat anti-chicken IgY (1:5,000, Thermo Fisher Scientific, cat. no. A11039). Western blots (Extended Data Fig. 2c,d) were performed two times.

### Immunostaining of paraffin-embedded foetal testis sections

#### Paraffin sections

Foetal testis samples were dissected at 13.5, 15.5, or 18.5 dpc, and fixed in 4% paraformaldehyde solution for 18, 20 and 22 h, respectively, at 4 °C before processing for paraffin embedding. The paraffin block was sectioned at 3 µm or 5 µm thickness, and sections were floated onto glass slides.

#### Immunostaining

Paraffin-embedded foetal testis sections were deparaffinized by xylene and gradient ethanol solutions. Heat-induced antigen retrieval was performed in 1× sodium citrate buffer (10 mM sodium citrate and 0.05% Tween 20, pH 6.0) by six cycles of heating to boiling point and cooling to room temperature for 2 min. Slides were gently rinsed in 1× PBS. Permeabilization was performed in PBST solution (0.5% Triton in PBS) at room temperature for 30 min. The slide was blocked in 10% goat serum, 1% BSA for 1 h at room temperature. Primary antibody incubation was carried out at 4 °C overnight according to dilution ratios as follows: rabbit anti-ZBTB43 antibody (Aviva Systems Biology, cat. no. ARP39048-P050) 1:100; rabbit anti-γH2AX (ABclonal, cat. no. AP0099) 1:200; mouse anti-Z22 (AbsoluteAntibody, cat. no. Ab00783-3.0) 1:10,000; mouse anti-DDX4 (Abcam, cat. no. ab27591) 1:100; rabbit anti-PGC7 (Abcam, cat. no. ab19878) 1:200; mouse anti-OCT3/4 (Santa Cruz Biotechnology, cat. no. sc-5279) 1:100. Slides were washed with 1× PBS before secondary antibody incubation. Secondary antibody reactions were carried out at room temperature, in the dark for 2 h according to dilution ratios as follows: anti-mouse-488 (Thermo Fisher Scientific, cat. no. A28175, 1:2,000), anti-rabbit-568 (Thermo Fisher Scientific, cat. no. A11011, 1:2,000). Slides were counterstained with DAPI at 1 mg ml^−1^, at room temperature for 10 min. Prolong Gold Antifade solution (Thermo Fisher Scientific, cat. no. P36934 or P36935 that contained DAPI) was applied to mount each slide before imaging. Each experiment was repeated twice using four to six sections of two different testis samples. Statistical analysis was performed using the Prism software by multiple non-paired two-sided *t*-tests (two-tailed) to obtain the *P* values. The *q* values are also provided in the source data with two-stage linear step-up procedure of Benjamini, Krieger and Yekuteli.

#### Microscopy

Images were taken by a Nikon A1plus-RSi Laser Scanning Confocal Microscope with 100× oil objectives.

### DNA–protein affinity sequencing assay

#### Affinity capture

His-MBP-ZBTB43 recombinant protein was purified from *E. coli* and stored in dialysis buffer (50 mM HEPES pH 7.4, 50% glycerol and 5 mM 2-mercaptoethanol) at −80 °C. Unmethylated genomic DNA was purified from mouse TKO ES cells (*Dnmt1*^−/−^ *Dnmt3a*^−/−^ *Dnmt3b*^−/−^) ES (clone19) RBRC no. AES0146 (ref. ^16^) with phenol–chloroform extraction and ethanol precipitation, and was sonicated to an average fragment size of 200 bp using a Covaris E220 Evolution ultrasound sonicator. When indicated, *M.Sss*I (NEB, cat. no. M0226M) was used to methylate the CpGs in fragmented mouse lung genomic DNA. His-MBP-ZBTB43 protein was pre-incubated with MBP-magnetic beads (NEB, cat. no. E8037S) at 4 °C for 90 min. To capture the ZBTB43 fraction, the fragmented mouse genomic DNA was mixed with the pre-incubated beads in a 200 µl reaction volume of binding buffer (10 mM Tris-HCl pH 7.8, 100 mM NaCl, 10 mM MgCl_2_, 0.05% NP40, 25 ng/µl BSA, 1 mM DTT, 0.05 mM ZnCl_2_) at 37 °C for 2 h. After incubation, the beads were washed with binding buffer 3 times to remove unbound DNA. The bound DNA was eluted in elution buffer (10 mM Tris–HCl pH 8.0, 10 mM EDTA, 500 mM NaCl, 1% SDS and 10 mM maltose) and collected by ethanol precipitation. Precipitated DNA was stored at −80 °C before library preparation.

#### Library preparation

The library was prepared with NEBNext Ultra II DNA Library Prep Kit (NEB cat. no. E7645S) according to the manufacturer’s instructions as described online (dx.doi.org/10.17504/protocols.io.wvgfe3w). Briefly, end preparation, adapter ligation and PCR amplification with index primers was performed using the captured DNA. The library was cleaned up twice before sequencing using 0.9× Ampure beads to remove the excess adaptors.

#### Deep sequencing and data analysis

Quality and quantity of the finished libraries were assessed using a combination of Agilent DNA High Sensitivity chip (Agilent Technologies) and Promega QuantiFluor dsDNA System (Promega), and 50 bp, paired-end sequencing was performed on an Illumina NovaSeq6000 sequencer using an S1, 100 bp sequencing kit (Illumina) to a minimum depth of 30 million reads per library. Base calling was done by Illumina RTA3, and output of NextSeq Control Software (NCS) v2.0 was de-multiplexed and converted to FastQ format with Illumina Bcl2fastq v1.9.0.

### Mutation screening

#### Vector construction

Oligonucleotides listed in Fig. 3a (Midland Certified Reagents) were annealed with their complementary strands, phosphorylated with T4 polynucleotide kinase and ATP, and inserted at an *Eco*RI(932)–*Sal*I(965) cassette between the promoter and the coding region of the *LacZ* gene of pUCNIM^7^. Clones resulting from the transformation of DH5α cells were screened by restriction analysis and verified by sequencing. Plasmid DNA was prepared from One Shot Stbl3 cells (Invitrogen, cat. no. C7373-03).

#### Z-DNA-induced mutagenesis of the *LacZ* gene in mammalian COS-7 cells

Mutagenesis was done as we described earlier^7^. COS-7 cells were transfected with the Z-DNA-forming plasmids, pUPZ1, pUPZ2 or the control plasmid pUCON, using GenePORTER (Genlantis) according to the manufacturer’s recommendations. After 48 h, the amplified plasmids were recovered by the method of Hirt^46^. After treatment with *Dpn*I to remove those plasmids that were not replicated in the COS-7 cells, the plasmids were transfected into DH5α cells or NEB Stable cells (New England BioLabs, cat. no. C3040H) to detect the *LacZ* mutants using a blue–white screen. Differences in mutation frequencies were analysed using two-tailed Student’s *t*-tests (unequal variance) from three independent experiments. DNA sequencing of the mutation reporter plasmids was carried out with the upstream sequencing primer 548: GGTGATGACGGTGAAAACCT, which was close to the non-B DNA insert, and in case of larger deletions using primer 201: CGTTTCTGGGTGAGCAAAA.

### EMSAs

#### ZBTB43 EMSA

His-MBP-ZBTB43-FL (full length) and GST-ZBTB43-ZF (ZF domain) and His-MBP-ZBTB43-BTB (BTB domain) were purified from *E. coli* against the His tag or GST tags, respectively. The 5′-FAM probes were synthesized by IDT. The probe sequences are provided in Supplementary Table 3. Briefly, 0.5 pmoles of FAM-labelled double-stranded DNA probe was incubated with 60 pmoles of ZBTB43 (FL) or 12.5 pmoles of ZBTB43 (ZF) or 12.5 pmoles of ZBTB43 (BTB) in 10 µl volume of binding buffer (10 mM Tris–HCl pH 7.8, 100 mM NaCl, 10 mM MgCl_2_, 0.05% NP40, 25 ng µl^−1^ BSA, 1 mM DTT, 0.05 mM ZnCl_2_ and 6.25 ng µl^−1^ sonicated unmethylated *E. coli* genomic DNA) at 37 °C for 2 h. Competition assay was carried out with 100× unlabelled competitor probe (50 pmoles) on ice for 20 min before adding the FAM-labelled probe.

#### Z-DNA EMSA

Z-DNA antibody (Z22) (AbsoluteAntibody cat. no. Ab00783-3.0) was used to detect Z-DNA by shifting the Z-DNA structure in EMSA gels using FAM-labelled double-stranded linear probes (CACG)_8_, and *Rps6kl1*. 0.5 pmoles of probe was induced by hexamine CoCl_3_ at 100 µM, 200 µM and 400 µM concentrations for 30 min at room temperature followed by incubation with 500 ng of Z22 antibody in 10 µl EMSA binding buffer (10 mM Tris–HCl pH 7.8, 0.05% NP40, 25 ng µl^−1^ BSA,1 mM DTT and 6.25 ng *E. coli* fragmented genomic DNA) for 30 min at room temperature. To titrate the specific affinity between the Z22 antibody and Z-DNA, 0.5 pmoles of the FAM-labelled consensus probe (CACG)_8_ was induced with 200 µM hexamine CoCl_3_ in 10 µl binding buffer for 30 min at room temperature and was incubated with 250 ng, 300 ng or 500 ng of Z22 antibody for 30 min at room temperature. Reactions were subjected to electrophoresis on a 4% native polyacrylamide gel in 0.5× TBE running buffer. Gel images were collected using the Alexa 488 channel of the Chemidoc MP imaging system.

### Preparation of circular double-stranded DNA probes

Topologically restrained circular DNA, CC and control CL and LL probes were prepared according to Zhang^21^.

#### Circulation of single-stranded DNA

Single-stranded DNA was synthesized by IDT with a phosphate at the 5′ position. The sequences of single-stranded DNAs and splint DNAs are listed in Supplementary Table 3. The cyclization of single-stranded DNA was carried out as follows. Briefly, the splint (960 pmoles) was dissolved in 120 µl of 0.2× T4 DNA ligase buffer (0.1 mM ATP, 2 mM MgCl_2_, 2 mM DTT and 8 mM Tris–HCl). The single-stranded linear DNA substrate (480 pmoles) and T4 ligase (20 U) was combined in 120 µl 0.1× T4 DNA ligase buffer and then separated into ten portions, and each portion was step by step added into the cyclization system every 20 min at room temperature. The reaction was continued at 4 °C for overnight with 2 U additional T4 ligase. Reaction was terminated by incubating it at 65 °C for 10 min. The remaining substrate DNA and linear byproducts were removed using exonuclease I (NEB, cat. no. M0293S) at 37 °C for 2 h and DNA was purified by phenol–chloroform extraction and ethanol precipitation. The single-stranded DNA circles were purified after gel electrophoresis as described earlier^21^.

#### Preparation of double-stranded circular DNA (CC)

The single-stranded circle and its complementary circle were combined at a final concentration of 4 µM in 1× annealing buffer (10 mM HEPES pH 7.5 and 10 mM MgCl_2_) and were annealed to generate a Z–B chimaera using the following annealing steps: 90 °C 5 min, cooling to 60 °C with a rate of 0.1 degree per second; 60 °C 10 min, cooling to 20 °C with a rate of 0.1 degree per second; and 20 °C 10 min. As B-DNA control, the double-stranded DNA was annealed in the same 1× annealing buffer using the same annealing steps either from two single-stranded linear DNA (LL) or from one single-stranded circle and one single-stranded linear DNA (CL-nicked). The nick in the CL-nicked circle was closed by ligation (CL-lig or simply CL).

#### EMSA of the CC DNA

To detect the formation of Z-DNA in the CC probe, a gel-shift assay was performed with the Z22 antibody (Absolute Antibody, cat. no. Ab00783-3.0). A total of 1.5 pmoles of the CC probe was incubated with different amounts (0.1 pmoles, 0.2 pmoles and 0.5 pmoles) of Z22 antibody in 10 µl reaction buffer (10 mM HEPES pH 7.5 and 10 mM MgCl_2_) at room temperature for 2.5 h. Reactions were subjected to electrophoresis in a 4% native polyacrylamide gel (pre-run for 1 h) in 0.5× TBE running buffer at room temperature for 2 h. The gel was stained with SYBR Green II (Thermo Fisher, S7594) in the dark at room temperature for 10 min before imaging. Gel images were collected using the Chemidoc MP imaging system.

### CC–CL competition

The probes CC and CL were combined at a 1:1 ratio, and 1.5 pmoles of the CC–CL combined probe was incubated with different amounts (3.75, 7.5, 12, 15, 21, 27, 30, 45, 60, 90, 120 and 150 pmoles) of ZBTB43(FL) in 8 µl volume of binding buffer (10 mM HEPES pH 7.5 and 10 mM MgCl_2_) at 37 °C for 2 min. Reactions were subjected to electrophoresis in a 4% native polyacrylamide gel (pre-run 1 h) in 0.5× TBE running buffer at room temperature for 3.5 h. The gel was stained with SYBR Green II (Thermo Fisher, S7594) in the dark at room temperature for 10 min before imaging. Gel images were collected using the ChemidocTMMP imaging system. Quantification was done by Fiji.

#### Restriction digestion of CC, CL and LL DNA

The respective restriction enzymes and expected fragment sizes after digesting linear DNA are listed below. Circular DNA in B conformation was expected to be linearized by these enzymes. We incubated 1.5 pmoles of probe DNA (CC, CL or LL) with 2 U (or 4 U when indicated) of *Hha*I (NEB, R0139S) restriction enzyme at 37 °C, *Bsiw*l (NEB, R0553S) at 55 °C or with *Tai*I (Thermo, ER1141) at 65 °C for 12 h. After incubation, reactions were subjected to electrophoresis in a 12% native polyacrylamide gel in 0.5× TBE running buffer at room temperature for about 2.5 h. The gel was stained with SYBR Green II (Thermo Fisher, S7594) in the dark at room temperature for 10 min before imaging. Gel images were collected using the Chemidoc MP imaging system.

### DNMT3A assay of CC, CL and LL DNA

Recombinant DNMT3A protein was purchased from Active Motif (cat. no. 31406). A total of 0.5 pmoles of DNA template (CC, CL or LL) was incubated together with 3 pmoles or 6 pmoles of DNMT3A as indicated, and 6,400 pmoles of SAM (B9003S, NEB) in 1× reaction buffer (20 mM HEPES pH 7.5, 50 mM KCl, 1 mM EDTA and 0.25 mg ml^−1^ BSA) at 37 °C for 4 h. Freshly thawed SAM (6,400 pmoles) was added every 1 h followed by pipetting up and down six times. DNA was purified by phenol–chloroform extraction and ethanol precipitation.

### Multiplex bisulfite sequencing of CC, CL and LL DNA

DNMT3A-methylated CC and CL probes were linearized before bisulfite conversion using *Sac*I restriction enzyme digestion, which cuts in the B-DNA part of CC and CL probes. DNA was purified by phenol–chloroform extraction and ethanol precipitation. The EZ DNA methylation kit (D5001, ZYMO) was used for the bisulfite conversion of linearized CC or CL probes and LL probes according to kit instructions. Multiplex PCR was performed with indexed primers and Zymo Taq hot start polymerase (E2002, ZYMO) to amplify the converted DNA. Amplified DNA fragments were resolved using a 1% agarose gel, and DNA was isolated from the gel slices using Monarch DNA Gel Extraction Kit (cat. no. T1020S). In total, 50 ng of each fragment was amplified with different index primers as listed in Supplementary Table 3 for Amplicon-EZ sequencing by GENEWIZ.

### CD spectroscopy

B-DNA and Z-DNA absorption spectra were measured between wavelengths 230 nm and 320 nm at the speed of 100 nm min^−1^ by Chirascan CD spectrometer (Applied Photophysics) (Fig. 4b) or by Jasco J-815 (Fig. 5 e–h) using a quartz cuvette (Hellma Analytics, art. no.100-1-40) with a path length of 1 mm. In the CoCl_3_ experiment, the baseline spectrum was calibrated for the reaction buffer (10 mM Tris–HCl pH 7.8 and 15 mM NaCl). For CD spectroscopy of B-DNA, 5 µg double-stranded linear (CACG)_8_ probe was dissolved in 200 µl reaction buffer. For CD spectroscopy of Z-DNA, 5 µg double-stranded linear (CACG)_8_ probe was reacted in 200 µl reaction buffer with hexamine CoCl_3_ (ref. ^47^) at molar ratios of 1:4,000 and 1:8,000 (probe: hexamine CoCl_3_). The Z-α domain of ADAR1 was purified as described earlier^48,49^. In the Z-α experiment, the baseline spectrum calibration was carried out for all reaction components without the DNA: 10 mM Tris–HCl pH 8.0, 50 mM NaCl, 2,200 pmoles (or 4,400 pmoles or 8,800 pmoles) of Z-α or Z-α’s storage buffer. In the ZBTB43 experiment, the baseline spectrum calibration was carried out for all reaction components without the DNA: 4,400 pmoles of ZBTB43(FL) or ZBTB43(FL)’s storage buffer. In the Z-α plus ZBTB43 experiment, the baseline spectrum calibration was carried out for all reaction components without the DNA: 2,200 pmoles (or 4,400 pmoles or 8,800 pmoles) of Z-α or Z-α’s storage buffer and 4,400 pmoles of ZBTB43(FL) or ZBTB43(FL)’s storage buffer. After baseline spectrum calibration, 5 μg (4.4 µl) double-stranded linear probe (CA)_16_ was added to 195.6 µl baseline calibration buffer to measure the CD spectrum of DNA. Each CD spectrum was presented as an average of three scans.

### 2D gel

#### Plasmid construction

Plasmids used for 2D gel electrophoresis were generated by PCR and were subcloned using TA cloning in pGEM-T vector (Promega, cat. no. A3600). The sequences were confirmed by Sanger sequencing. The insertion for pGEM-Rps6kl1 contains the central 417 bases from ZBTB43 affinity peak in the intron of the mouse *Rps6kl1* gene. The insertion for pGEM-Ago2(I2) contains 417 bases from exon 4 of the *Ago2* gene, which is not recognized by the ZBTB43 protein. The sequences of the plasmid inserts are listed in Supplementary Table 3.

#### Preparation of plasmid topoisomers

Topoisomerase I (Thermo Fisher, cat. no. 38042024,) was used to relax the plasmid DNA to different extents depending on the ethidium bromide (Sigma-Aldrich, cat. no. E1510-10 ml) concentrations (0 to 24 µg ml^−1^) in its 1× supplied reaction buffer (50 mM Tris–HCl, pH 7.8, 50 mM NaCl, 10 mM MgCl_2_, 0.5 mM DTT, 0.1 mM EDTA and 30 µg ml^−1^ BSA) at 37 °C for 2 h. Phenol–chloroform extraction was used to remove ethidium bromide from the plasmid DNA. Different levels of negative supercoiling counteracted the torsional stress upon removal of the intercalator. Ethanol precipitation was used to collect the DNA.

#### 2D gel electrophoresis

In total, 625 ng of DNA was combined from a series of topoisomerase reactions. Topoisomers were resolved by gel electrophoresis in a 1.5% agarose gel with 0.5× TBE running buffer. After the first dimension of electrophoresis, the gel was soaked in 10 µg ml^−1^ chloroquine (Sigma, cat. no. C6628-25G) at room temperature in the dark for 6 h. The gel was then turned 90 degrees and was electrophoresed in the second dimension in 0.5× TBE containing 10 µg ml^−1^ chloroquine. The gel was stained with 10 µg ml^−1^ ethidium bromide in the dark at room temperature for 1 h and imaged using a Chemidoc MP imaging system.

### ZBTB43 ChIP–seq

#### Purification of foetal MGCs

To obtain foetuses for germ cell purification, wild-type C57Bl/6J or TgOG2 (ref. ^15^) females were crossed with TgOG2 males. Testicles were dissected from the resulting male foetuses at 15.5 dpc, and were dissociated to single-cell suspension using trypsin digestion and trituration as described earlier^15^. Up to eight testes were digested in 150 µl 0.25% trypsin (including 0.5% BSA) at 36 °C for 15 min, tapping gently every 5 min. The trypsin reaction was stopped by adding 450 µl 20% FBS in M2 medium (Sigma, cat. no. M7167). After filtering, germ cells were purified by flow cytometry using a MoFlo Astrios (Beckman Coulter) sorter with a 100 µm nozzle at 25 psi. The acquisition software is Summit v 6.3. About 90% of the total population was gated as healthy cells, then single cells were positively separated from doublets and triplets using both forward and side scatter plots. Single EGFP^+^ cells were then positively gated for EGFP expression.

#### ChIP–seq of foetal germ cells

ChIP was performed according to Singh^26^. For the 100,000 ZBTB43 ChIP and 100,000 IgG ChIP, 100,000 FACS purified EGFP^+^ germ cells were pelleted down at 750*g* (3,000 rpm) for 10 min at room temperature. Crosslinking was done by adding 500 µl of 1% formaldehyde in 1× PBS, followed by rotating on a platform gently at room temperature for 10 min. Then, 25 µl of 1.25 M glycine was added into each sample to stop crosslinking. Germ cells were pelleted at 750*g* for 10 min at room temperature. Cell pellets were suspended in 100 µl of ChIP lysis buffer^26^ and were snap-frozen in liquid nitrogen and kept at −80 °C until used for sonication. Before sonication, 20 µl of 6.5× shearing buffer (65 mM Tris–HCl pH 8.0, 6.5 mM EDTA and 0.65% SDS) was added to each sample. Sonication was carried out using a Covaris E220 Evolution ultrasound sonicator (PIP: 105; Duty Factor: 2; CPB: 200; Time: 12 min). For the 500,000 ZBTB43 ChIP sample or 500,000 IgG sample, crosslinking was done on combined batches of frozen prospermatogonia in 500 µl of 1% formaldehyde in 1× PBS. Reaction was stopped by adding 50 µl of 1.25 M glycine. Then, 100 µl of 6.5× shearing buffer was added to each sample. Sonication was carried out in five tubes. After sonication, 1/10 of the sheared chromatin was taken out for ‘input’. The remaining 9/10 of the sample was diluted tenfold with ChIP dilution buffer containing 4 µl ml^−1^ 50× PIC (Roche, cat. no. 11697498001), and 1 µg of the anti-ZBTB43 antibody (Aviva Systems Biology, cat. no. ARP39048-P050), or the control rabbit-IgG (Abcam, cat. no. ab171870), was added per 100,000 cells-worth chromatin and incubated overnight at 4 °C. The complex was washed, and the DNA was eluted as described earlier^26^. The captured DNA fraction was purified using phenol–chloroform extraction and ethanol precipitation and was stored at −80 °C until library preparation.

#### Library preparation

The library was prepared with NEBNext Ultra II DNA Library Prep Kit (New England Biolabs, cat. no. E7645S) according to the manufacturer’s protocol as described online (dx.doi.org/10.17504/protocols.io.wvgfe3w). Briefly, end preparation, adapter ligation and PCR amplification with index primers were performed using the captured DNA. The library was cleaned up before sequencing using 1× Ampure beads twice to remove the excess adaptors.

#### Deep sequencing

Quality and quantity of the finished libraries were assessed using a combination of Agilent DNA High Sensitivity chip (Agilent Technologies), QuantiFluor dsDNA System (Promega), and Kapa Illumina Library Quantification qPCR assays (Kapa Biosystems), and 150 bp paired-end sequencing was performed on an Illumina NovaSeq sequencer using a 150 bp high-output sequencing kit (v2) (Illumina) to a minimum depth of 40 million reads per library. Base calling was done by Illumina NextSeq Control Software (NCS) v2.0, and output of NCS was de-multiplexed and converted to FastQ format with Illumina Bcl2fastq v1.9.0.

### Genome-wide mapping of DNA methylation by MIRA–seq

#### Sperm collection by swim-up and DNA preparation

Adult male mice were killed at 3 months of age by CO_2_ asphyxiation. The cauda epididymis was dissected and cleaned from fat tissue in 1× PBS under microscope. Caudae were placed into 1 ml of M16 buffer, nicked with a blade three times and transferred with the buffer into a round-bottom tube. Then, 4 ml of additional M16 buffer was gently layered over the caudae. Samples were incubated, lid open, in a cell culture incubator at 37 °C with 5% CO_2_ for 2 h. The top 4 ml was transferred into a 15 ml tube without touching the bottom, and the cell number was counted. The motile spermatozoa were pelleted down at 835*g* (3,000 rpm) for 6 min. The sperm pellet was then suspended in 1 ml sperm lysis buffer (0.1 M DTT and 2 mg ml^−1^ proteinase K in 10 mM Tris–HCl pH 8.0, 10 mM EDTA and 1% SDS) per cauda and was incubated at 55 °C overnight. DNA was isolated with phenol–chloroform extraction and ethanol precipitation and was sonicated to an average fragment size of 200 bp using a Covaris E220 Evolution ultrasound sonicator.

#### GST-MBD2b and His-MBD3L1 preparation

MIRA–seq was carried out according to Jung^29^. GST-MBD2b and His-MBD3L1 was purified from *E. coli* against GST and His tags, respectively. Both proteins were stored in salt-dialysis buffer (50 mM HEPES pH 7.4, 50% glycerol, 5 mM 2-mercaptoethanol and 150 mM NaCl) at −20 °C for no more than 6 months.

#### MIRA reaction

MIRA proteins, 1 µg of GST-MBD2b and 1 µg His-MBD3L1, and the MagneGST beads (Promega, cat. no. V8611) were pre-blocked with 500 ng unmethylated *E.Coli* genomic DNA in 1× MIRA binding buffer (10 mM Tris–HCl pH 7.9, 50 mM NaCl, 1 mM DTT, 10 mM MgCl_2_ and 0.1% Triton-X-100) at 4 °C for 20 min. Then, 500 ng fragmented sperm DNA was combined with the pre-blocked MBD2b and MBD3L1 proteins, and the binding reaction was carried out at 4 °C, overnight with rotation. On the next day, 5 µl pre-blocked MagneGST beads was added to each MIRA reaction and the bound fraction was captured by incubating at 4 °C for 1 h with rotation. The beads were collected using a magnetic stand. They were washed three times using 900 µl prechilled MIRA wash buffer (10 mM Tris–HCl pH 7.5, 400 mM NaCl and 0.1% Triton-X-100). After washing, the beads were captured using a magnetic stand, DNA was collected by adding 500 µl buffer PB from the Qiagen PCR purification kit (cat. no. 28105) and DNA was isolated according to the kit’s instructions.

#### Deep sequencing

Libraries were prepared by the VAI Genomics Core from 2.5 ng of input material using the KAPA Hyper Prep Kit (v5.16) (Kapa Biosystems). Before PCR amplification, end-repaired and A-tailed DNA fragments were ligated to Bioo Scientific NEXTflex Adapters (Bioo Scientific). Quality and quantity of the finished libraries were assessed using a combination of Agilent DNA High Sensitivity chip (Agilent Technologies), QuantiFluor dsDNA System (Promega) and Kapa Illumina Library Quantification qPCR assays (Kapa Biosystems), and 75 bp, single-end sequencing was performed on an Illumina NextSeq 500 sequencer using a 75 bp mid-output sequencing kit (v2) (Illumina). Base calling was done by Illumina NextSeq Control Software (NCS) v2.0, and output of NCS was de-multiplexed and converted to FastQ format with Illumina Bcl2fastq v1.9.0.

### Bisulfite sequencing of genomic DNA

#### Bisulfite conversion

The EZ DNA methylation kit (ZYMO, cat. no. D5001) was used to convert 500 ng sonicated sperm DNA according to the manufacturer’s instructions. In total, 2,000 FACS-sorted male foetal germ cells were lysed with proteinase K solution and converted with EZ DNA methylation kit (ZYMO, cat. no. D5001) according to the manufacturer’s instructions.

#### Nested PCR and ligation

Two rounds of PCR were performed using nested primers as listed in Supplementary Table S3, and Zymo Taq hot start polymerase (ZYMO, cat. no. E2002) to amplify the converted DNA. The purified PCR was ligated into pGEM-T vector (Promega, cat. no. A3600). Sanger sequencing of 15 independent colonies per region was performed by GENEWIZ.

#### Data analysis of the bisulfite sequencing

QUMA online software^50^ was used for methylation analysis. Colonies that had identical pattern of the occasional incomplete conversion were excluded from the displays.

### Bioinformatics

#### MIRA–seq and affinity-seq analysis

Reads were aligned to the mm10 genome using bwa mem v0.7.17 (ref. ^51^) with default parameters for MIRA–seq and affinity-seq. Peaks were called for affinity-seq samples using MACS2 callpeak v2.2.7.1 (ref. ^52^) with default parameters, combining duplicate samples and using the corresponding MBP background samples as controls. Differentially methylated regions were called in MIRA–seq using MEDIPS^53^ with an extension size of 300 bp and window size of 100 bp. Differentially methylated windows with an adjusted *P* value <0.05 were split by the log fold change direction. Adjacent windows were merged using bedtools merge v2.29.2 (ref. ^54^), with the parameter ‘-d 1’.

#### ZBTB43 ChIP–seq analysis

Reads were trimmed using Trim Galore v0.6.0 (https://github.com/FelixKrueger/TrimGalore)^55^, then were aligned to the mm10 genome using bwa mem v0.7.17 (ref. ^51^), using default parameters for both steps. Duplicate alignments were marked using SAMBLASTER v0.1.24 (ref. ^56^) with the parameter ‘–addMateTags’. Alignments were filtered using samtools view v1.9 (ref. ^57^) with the parameters ‘-q 30 -F 2828 -f 2’ and removing mitochondrial alignments. Peaks were called using MACS2 v 2.2.7.1 (ref. ^52^) with the parameters ‘-f BAMPE -g mm -q 0.05–keep-dup 1’ and the corresponding input or IgG samples as control. Peaks overlapping ENCODE blacklist v2^58^ regions were removed using bedtools intersect v2.29.2 (ref. ^54^) with the ‘-v’ option.

### Visualization and peak analysis

To count overlaps between peak sets, peaks were imported into R as GenomicRanges^59^. We noticed 328 of the predicted ZDNA regions were exact duplicates, so we removed these using the unique() function. All peaks overlapping ENCODE blacklist v2 regions were removed using subsetByOverlaps() with ‘invert=TRUE’, except the ZBTB43 ChIP–seq peaks, which were already blacklist-filtered as described above. Peaks on unplaced contigs were removed using keepStandardChromosomes() with ‘pruning.mode = “coarse”’. Peak overlaps were identified and plotted in Venn diagrams using the makeVennDiagram function from the ChIPpeakAnno v3.26.4 package^60^ with the parameter ‘connectedPeaks = “merge”’. To subset peaksets for overlap with ZBTB43 ChIP–seq peaks for plotting heatmaps, findOverlaps() was used to find overlaps between the ZBTB43 ChIP–seq peaks and the relevant peakset. Peaks were annotated with the nearest gene using ChIPseeker v1.28.3 (ref. ^61^), based on gene annotations in TxDb.Mmusculus.UCSC.mm10.knownGene v3.10.0 and org.Mm.eg.db v3.13.0, and with the TSS region (promoter region) set to −3,000 to 0. Pie charts were plotted with plotAnnoPie() from ChIPseeker.

DeepTools v3.4.3 (ref. ^62^) was used to generate bigWig files and coverage heatmaps. For single-sample bigWig files, bamCoverage was run with the parameters ‘–binSize 10–normalizeUsing “CPM”–samFlagInclude 64–extendReads’ for paired-end samples. For single-end samples (MIRA–seq), the last two parameters were replaced with ‘–extendReads 200’. For bigWig files comparing immunopreciptation signals obtained using specific anti-ZBTB43 antibody to nospecific anti-IgG antibody, bamCompare was run with the parameters ‘–extendReads–binSize 10–samFlagExclude 1024–samFlagInclude 64’ and specifying the ENCODE blacklist v2 (ref. ^58^) using ‘–blackListFileName’. The input BAM files were as described in the above sections, except the affinity-seq alignments were additionally filtered, as described for the ZBTB43 ChIP–seq alignments, for the heatmaps involving ZBTB43 ChIP–seq peaks for consistency. To create the heatmaps, the computeMatrix reference-point function was used with the parameters, ‘–referencePoint “center”–binSize 10 -a 1000 -b 1000’. Outputs from computeMatrix were plotted using the plotHeatmap function. Plotted regions were blacklist-filtered beforehand as described for the Venn diagrams.

Bisulfite amplicon-seq analysis: Reads were trimmed using Trim Galore v0.6.0 (https://github.com/FelixKrueger/TrimGalore)^55^ with default settings to remove low-quality bases and Illumina adapters. De-multiplexing was done using CutAdapt v3.2 (ref. ^55^). The 6-mer barcode for each sample present in a multiplexed library was specified using the anchored 5′ adapter option (‘-g sampleID = ^barcode’), requiring no mismatches and no indels (‘-e 0–no-indels’). As CutAdapt looks for the barcode in R1 only, CutAdapt was run twice. In the first round, R1 and R2 were specified in the correct order. To account for reads where the barcode adapter was on R2, unmatched reads from the first CutAdapt run were processed with CutAdapt again using the same settings except R1 and R2 were specified in reverse (R1 as R2 and vice versa). To remove the remaining, non-barcode adapter sequence, CutAdapt was run once more for each of the two rounds of de-multiplexing, specifying R1 and R2 in the same order as the de-multiplexing step, and using ‘-g PASS = ^TTAAGGTTGGTTTGTGTTAGA’ with default parameters for mismatches and indels. This step also discards reads without the barcode sequence, and thus likely to be erroneously assigned to a particular sample. Finally, the R1 and R2 reads from the two rounds of de-multiplexing were concatenated together for each sample.

Reads were aligned to the corresponding target sequence using Bismark v0.23.0 (ref. ^63^) with the parameters ‘–non_directional’ and ‘–local’. Per-CpG methylation percentages were obtained using the ‘bismark_methylation_extractor’ script in Bismark with the parameters ‘–bedGraph’ and ‘–paired-end’.

### Statistics and reproducibility

Statistically significant changes (*P value) of different features was calculated by multiple twp-sided *t*-tests using Prism. In addition, we provide the *q* values in the source data files to Fig. 6d and Extended Data Figs. 6c–e and 8f–i after adjustment for multiple comparisons using the Benjamini–Hochberg–Yekutieli correction. Affinity-seq and ChIP–seq peaks (Supplementary Tables 1, 2 and 4) were called using the ‘callpeak’ command in MACS2. Briefly, MACS2 models the background (based on the control sample) read fragment distribution in windows across the genome using a Poisson distribution. *P* values are calculated for enrichment of read fragments in the enriched/treatment sample compared with the expected number of reads for a given genomic region. MACS2 also corrects for multiple testing using the Benjamini–Hochberg–Yekutieli correction to calculate *q* values. No statistical method was used to pre-determine sample size. The experiments were not randomized. The investigators were not blinded to allocation during experiments or outcome assessment.

Immunostaining assays, mutagenesis assays and biochemical assays were performed three times using three biologically independent replicates unless specified in the legends. For figure panels Figs. 1e and 2a (BTB domain), 3d,f,g, and 5e–h, experiments were performed once. For figure panels Figs. 1f, 2a (ZBTB43FL and ZF domain), 5a–d, 6e,f and 8a, experiments were performed twice. Deep sequencing assays, such as affinity-seq (Figs. 2c,e,f and Fig. 7a,f,g), ChIP–seq (Fig. 7a,f,g) and MIRA–seq (Fig. 8b,d), assays were performed once with biologically independent duplicates. Quantification and statistics were derived from one experiment unless specified in the legends.

### Reporting summary

Further information on research design is available in the Nature Research Reporting Summary linked to this article.

## Online content

Any methods, additional references, Nature Research reporting summaries, source data, extended data, supplementary information, acknowledgements, peer review information; details of author contributions and competing interests; and statements of data and code availability are available at 10.1038/s41556-022-00941-9.

## Supplementary information

Reporting Summary

Supplementary Table Supplementary Table 1. ZBTB43 affinity-seq peaks in fully methylated genomic DNA (SssI). Supplementary Table 2. ZBTB43 affinity-seq peaks in unmethylated genomic DNA (TKO). Supplementary Table 3. Oligonucleotides and probes. Supplementary Table 4. ZBTB43 ChIP–seq peaks in 15.5 dpc prospermatogonia. Supplementary Table 5. DMRs in *Zbtb43* mutant sperm (MIRA–seq).

## Data Availability

The genomics data have been deposited to GEO database and can be accessed in the link https://www.ncbi.nlm.nih.gov/geo/query/acc.cgi?acc=GSE200729 under superseries GSE200729. Source data are provided with this paper. All other data supporting the findings of this study are available from the corresponding author on reasonable request.

## Source data

Source Data Fig. 2 Unprocessed blots and/or gels.
Source Data Fig. 3 Statistical source data.
Source Data Fig. 4 Statistical source data.
Source Data Fig. 4 Unprocessed blots and/or gels.
Source Data Fig. 5 Unprocessed blots and/or gels.
Source Data Fig. 6 Statistical source data.
Source Data Extended Data Fig. 2 Unprocessed blots and/or gels.
Source Data Extended Data Fig. 6 Statistical source data.
Source Data Extended Data Fig. 8 Statistical source data.
