# Supplementary material for: Z-DNA is remodelled by ZBTB43 in prospermatogonia to safeguard the germline genome and epigenome
Source: Nat Cell Biol. 2022 Jul 4;24(7):1141–53. doi: 10.1038/s41556-022-00941-9 (PMC9276527; doi:10.1038/s41556-022-00941-9)
Supplement: Supplementary file 1 — Reporting Summary [file 41556_2022_941_MOESM1_ESM.pdf]

nature research

Corresponding author(s): Piroska E Szabó

Last updated by author(s): April 10, 2022

Reporting Summary

Nature Research wishes to improve the reproducibility of the work that we publish. This form provides structure for consistency and transparency in reporting. For further information on Nature Research policies, see our Editorial Policies and the Editorial Policy Checklist.

Statistics

For all statistical analyses, confirm that the following items are present in the figure legend, table legend, main text, or Methods section.

n/a	Confirmed	
☐	☒	The exact sample size (*n*) for each experimental group/condition, given as a discrete number and unit of measurement
☐	☒	A statement on whether measurements were taken from distinct samples or whether the same sample was measured repeatedly
☐	☒	The statistical test(s) used AND whether they are one- or two-sided<br>*Only common tests should be described solely by name; describe more complex techniques in the Methods section.*
☒	☐	A description of all covariates tested
☒	☐	A description of any assumptions or corrections, such as tests of normality and adjustment for multiple comparisons
☐	☒	A full description of the statistical parameters including central tendency (e.g. means) or other basic estimates (e.g. regression coefficient) AND variation (e.g. standard deviation) or associated estimates of uncertainty (e.g. confidence intervals)
☐	☒	For null hypothesis testing, the test statistic (e.g. *F*, *t*, *r*) with confidence intervals, effect sizes, degrees of freedom and *P* value noted<br>*Give P values as exact values whenever suitable.*
☒	☐	For Bayesian analysis, information on the choice of priors and Markov chain Monte Carlo settings
☒	☐	For hierarchical and complex designs, identification of the appropriate level for tests and full reporting of outcomes
☒	☐	Estimates of effect sizes (e.g. Cohen's *d*, Pearson's *r*), indicating how they were calculated

Our web collection on statistics for biologists contains articles on many of the points above.

Software and code

Policy information about availability of computer code

Data collection	Base calling of genomics data was done by Illumina NextSeq Control Software (NCS) v2.0 and output of NCS was demultiplexed and converted to FastQ format with Illumina Bcl2fastq v1.9.0.

Data analysis	Bcl2fastq v1.9.0<br>Bismark v0.23.0<br>bwa v0.7.17<br>ChIPpeakAnno v3.26.4<br>ChIPseeker v1.28.3<br>CutAdapt v3.2<br>DeepTools v3.4.3<br>MACS2 v2.2.7.1<br>MEDIPS<br>bedtools v2.29.2<br>Microsoft Excel 16.42<br>NCS v2.0<br>Prism v9.3.1<br>QUMA<br>R 4.1.0<br>SAMBLASTER v0.1.24<br>samtools v1.9<br>Summit v6.3<br>Trim Galore v0.6.0

For manuscripts utilizing custom algorithms or software that are central to the research but not yet described in published literature, software must be made available to editors and reviewers. We strongly encourage code deposition in a community repository (e.g. GitHub). See the Nature Research guidelines for submitting code & software for further information.

Data

Policy information about availability of data

All manuscripts must include a data availability statement. This statement should provide the following information, where applicable:

- Accession codes, unique identifiers, or web links for publicly available datasets
- A list of figures that have associated raw data
- A description of any restrictions on data availability

Data availability: The genomics data have been deposited to GEO database under superseries GSE200729, and can be accessed in the link https://www.ncbi.nlm.nih.gov/geo/query/acc.cgi?acc=GSE200729.

Field-specific reporting

Please select the one below that is the best fit for your research. If you are not sure, read the appropriate sections before making your selection.

☒ Life sciences ☐ Behavioural & social sciences ☐ Ecological, evolutionary & environmental sciences

For a reference copy of the document with all sections, see nature.com/documents/nr-reporting-summary-flat.pdf

Life sciences study design

All studies must disclose on these points even when the disclosure is negative.

Sample size	No statistical method was used to predetermine sample size. Sample sizes were chosen based on practical considerations and norms in the field of the type of tudies.
Data exclusions	Duplicated reads or multiply mapped reads were excluded where noted in the methods. Peaks and regions on unplaced contigs or overlapping the ENCODE blacklist v2 were removed where noted.
Replication	Immunostaining, and in vitro biochemistry experiments (EMSA, mutation assays, 2D gels, Circular dichroism assays, TOPO1 assays, DNMT3A methylation, CC-CL-LL restriction digestion, etc.) were carried out at least two times and with the same outcome. Affinity-seq, MIRA-seq and ChIP-seq experiments were carried out in biological duplicates and the results were consistent between duplicates.
Randomization	Randomization was not applied, as we did not analyze the consequence of a treatment.
Blinding	Blinding was not applied, because we used mouse genetic mutants whose genotype was known to us. This does not affect our findings, as our experiments were not based on subjective measurements.

Reporting for specific materials, systems and methods

We require information from authors about some types of materials, experimental systems and methods used in many studies. Here, indicate whether each material, system or method listed is relevant to your study. If you are not sure if a list item applies to your research, read the appropriate section before selecting a response.

Materials & experimental systems

n/a	Involved in the study	
☐	☒	Antibodies
☐	☒	Eukaryotic cell lines
☒	☐	Palaeontology and archaeology
☐	☒	Animals and other organisms
☒	☐	Human research participants
☒	☐	Clinical data
☒	☐	Dual use research of concern

Methods

n/a	Involved in the study	
☐	☒	ChIP-seq
☐	☒	Flow cytometry
☒	☐	MRI-based neuroimaging

Antibodies

Antibodies used

Primary antibodies used for Western blot:
ZBTB43 (Aviva Systems Biology, Cat. No. ARP39048-P050, Lot QC19506-40743)
LacZ (Abcam, Cat. No. ab9361, Lot. GR3232668);
GAPDH (Abclonal, Cat. No. AC035, Lot unknown);

Secondary antibodies for Western blot:
HRP goat anti-rabbit IgG (Active Motif, Cat. No. 15015, Lot 4318009);
Alexa Fluor 488 goat anti-chicken IgY (Thermo Fisher SCIENTIFIC, Cat. No. A11039 Lot 2078383)

Primary antibodies used for immunohistochemistry:
Rabbit anti-ZBTB43 (Aviva Systems Biology, Cat. No. ARP39048-P050, Lot QC19506-40743)
Rabbit anti-gammaH2AX (ABclonal, Cat. No. AP0099, Lot 210140101)
Mouse anti-Z-DNA Z22 (AbsoluteAntibody, Cat. No. Ab00783-3.0, Lot T190B320)
Mouse anti-DDX4 (Abcam, Cat. No. ab27591, Clone mAbcam27591, Lot GR3401601-2)
Rabbit anti-PGC7 (Abcam Cat. No. ab19878, Lot unknown)
Mouse anti-OCT3/4 (Santa Cruz Biotechnology, Cat No. sc-5279, Lot D1317)

Secondary antibodies used for immunohistochemistry:
anti-mouse-488 (Thermo Fisher SCIENTIFIC, Cat. No. A28175)
anti-rabbit-568 (Thermo Fisher SCIENTIFIC, Cat. No. A11011)

Antibody for EMSA:
Z-DNA antibody (Z22) (AbsoluteAntibody Cat. No. Ab00783-3.0, Lots T190B320 and T2016B48)

Antibody used for ChIP-seq:
Rabbit anti-ZBTB43 antibody (Aviva Systems Biology Cat. No: ARP39048-P050, Lot QC19506-40743)
Rabbit anti-rabbit-IgG (Abcam Cat No: ab171870. Lot GR3228514-3)

Validation

The most critical primary antibodies of this study were validated by independent methods:
Z-DNA antibody (Z22) (Aviva Systems Biology, Cat. No. Ab00783-3.0): Validated by EMSA using cobalt-induced Z-DNA and immunostaining of curaxin-induced Z-DNA in mouse fibroblasts.
ZBTB43 (antibodies-online, Cat. No. ARP39048-P050): Validated by Western blot of Zbtb43-/- and wild type mouse kidney. Validated also by immunostating of fetal Zbtb43-/- and wild type mouse testis.

Validation by manufacturers:

Primary antibodies used for Western blot:
ZBTB43 (Aviva Systems Biology, Cat. No. ARP39048-P050); host, rabbit; application, Western blotting
LacZ (Abcam, Cat. No. ab9361); host, chicken; application, IHC-FrFl, IHC-FoFr, ICC/IF, IHC-Fr, IHC-P
GAPDH (Abclonal, Cat. No. AC035); host, mouse; application, Western blotting

Primary antibodies used for immunohistochemistry:
Rabbit anti-ZBTB43 antibody (Aviva Systems Biology, Cat. No. ARP39048-P050); host, rabbit, application, Western blotting
Rabbit anti-gammaH2AX (ABclonal, Cat. No. AP0099) host, rabbit, application, IF
Mouse anti Z-DNA Z22 (AbsoluteAntibody, Cat. No. Ab00783-3.0); host, mouse; application, gel retardation assay, SPR, ELISA, IF
Mouse anti-DDX4 (Abcam, Cat. No. ab27591); host, mouse; applications, ICC/IF, IHC-P, WB
Rabbit anti-PGC7 (Abcam Cat. No. ab19878); host, rabbit; application, IHC-Fr, WB, ICC/IF
Mouse anti-OCT3/4 (Santa Cruz Biotechnology, Cat No. sc-5279); host, mouse; application, WB, IP, IF, IHC(P), FCM and ELISA

Antibody used for EMSA:
Z-DNA antibody (Z22) (AbsoluteAntibody Cat. No. Ab00783-3.0); host, mouse; application, gel retardation assay, SPR, ELISA, IF

Antibody used for ChIP-seq:
Rabbit anti-ZBTB43 antibody (Aviva Systems Biology Cat. No: ARP39048-P050); host, rabbit; application, Western blotting

Rabbit anti-rabbit-IgG (Abcam Cat No: ab171870); host, rabbit: application, WB, ChIP, Flow Cytometry

Eukaryotic cell lines

Policy information about cell lines

Cell line source(s)	TKO ES cells (Dnmt1-/-Dnmt3a-/-Dnmt3b-/-) ES (clone19) RBRC No.: AES0146 provided by Masaki Okano, Ph.D. (Kumamoto University).<br>COS-7 cells (ATCC)
Authentication	Neither of these cell lines were authenticated.
Mycoplasma contamination	The cells were not tested for Mycoplasma contamination.
Commonly misidentified lines (See ICLAC register)	The cell lines are not listed in the commonly misidentified list.

Animals and other organisms

Policy information about studies involving animals; ARRIVE guidelines recommended for reporting animal research

Laboratory animals	The Zbtb43+/- heterozygous mouse C57BL/6N-Zbtb43tm1b(KOMP)Mbp/J was obtained from the Jackson Laboratory, and kept in the C57BL/6N genetic background. A sub-line was created by back-crossing to JF1/Ms 1 mice. To obtain wild type prospermatogonia for ChIP-seq by FACS, C57BL/6J females (~2 months old) were crossed with B6;CBA-Tg(Pou5f1-EGFP)2Mnn 2 (TgOG2) transgenic males (2-12 months old) and male fetuses were collected at 15.5 dpc. Zbtb43+/-; TgOG2 females (~2 months old) were crossed with Zbtb43 +/-; TgOG2 transgenic males (2-12 months old) to obtain mutant and wild type fetuses at 18.5 dpc, for collecting prospermatogonia by FACS. Adult Zbtb43-/- and wild type males at 3 months of age were uses for sperm collection. Mice were kept on a 12-hour light/dark cycle, 7am to 7pm at ambient room temperature of 72 degrees F, and at humidity of 30-70%, monitored through the building control management system.
Wild animals	No wild animals were used in this study.
Field-collected samples	The study did not involve field-collected samples.
Ethics oversight	All animal experiments were performed according to the National Institutes of Health Guide for the Care and Use of Laboratory animals, with Institutional Care and Use Committee-approved protocols at Van Andel Institute (VAI).

Note that full information on the approval of the study protocol must also be provided in the manuscript.

ChIP-seq

Data deposition

☒ Confirm that both raw and final processed data have been deposited in a public database such as GEO.

☒ Confirm that you have deposited or provided access to graph files (e.g. BED files) for the called peaks.

Data access links<br>*May remain private before publication.*	https://www.ncbi.nlm.nih.gov/geo/query/acc.cgi?acc=GSE200729.
Files in database submission	Affinity-seq samples:<br><br>Affinity-seq MBP background SssI methylated lung DNA replicate 1<br>Affinity-seq MBP background SssI methylated lung DNA replicate 2<br>Affinity-seq MBP background unmethylated TKO ES DNA replicate 1<br>Affinity-seq MBP background unmethylated TKO ES DNA replicate 2<br>Affinity-seq MBP background Zbtb43-/- hypomethylated sperm DNA<br>Affinity-seq MBP background Zbtb43+/+ normally methylated sperm DNA<br>Affinity-seq MBP-ZBTB43 SssI methylated lung DNA replicate 1<br>Affinity-seq MBP-ZBTB43 SssI methylated lung DNA replicate 2<br>Affinity-seq MBP-ZBTB43 unmethylated TKO ES DNA replicate 1<br>Affinity-seq MBP-ZBTB43 unmethylated TKO ES DNA replicate 2<br>Affinity-seq MBP-ZBTB43 Zbtb43-/- hypomethylated sperm DNA<br>Affinity-seq MBP-ZBTB43 Zbtb43+/+ normally methylated sperm DNA<br><br>Affinity-seq Raw files:<br>55VectorSSSi1_L000_R1_001.fastq.gz<br>6VectorSSSi2_L000_R1_001.fastq.gz<br>1VectorTKN1_L000_R1_001.fastq.gz<br>2VectorTKN2_L000_R1_001.fastq.gz<br>8VectorMuTSperm_L000_R1_001.fastq.gz

7VectorWTSperm_L000_R1_001.fastq.gz
13ZBTB43SSSi1_L000_R1_001.fastq.gz
14ZBTB43SSSi2_L000_R1_001.fastq.gz
9ZBTB43TKN1_L000_R1_001.fastq.gz
10ZBTB43TKN2_L000_R1_001.fastq.gz
16ZBTB43MuTSperm_L000_R1_001.fastq.gz
15ZBTB43WTSperm_L000_R1_001.fastq.gz
5VectorSSSi1_L000_R2_001.fastq.gz
6VectorSSSi2_L000_R2_001.fastq.gz
1VectorTKN1_L000_R2_001.fastq.gz
2VectorTKN2_L000_R2_001.fastq.gz
8VectorMuTSperm_L000_R2_001.fastq.gz
7VectorWTSperm_L000_R2_001.fastq.gz
13ZBTB43SSSi1_L000_R2_001.fastq.gz
14ZBTB43SSSi2_L000_R2_001.fastq.gz
9ZBTB43TKN1_L000_R2_001.fastq.gz
10ZBTB43TKN2_L000_R2_001.fastq.gz
16ZBTB43MuTSperm_L000_R2_001.fastq.gz
15ZBTB43WTSperm_L000_R2_001.fastq.gz

Affinity-seq Processed files:
5VectorSSSi1_filt_alns_keepdups.bw
6VectorSSSi2_filt_alns_keepdups.bw
1VectorTKN1_filt_alns_keepdups.bw
2VectorTKN2_filt_alns_keepdups.bw
8VectorMuTSperm.bw
7VectorWTSperm.bw
13ZBTB43SSSi1_filt_alns_keepdups.bw
14ZBTB43SSSi2_filt_alns_keepdups.bw
9ZBTB43TKN1_filt_alns_keepdups.bw
10ZBTB43TKN2_filt_alns_keepdups.bw
16ZBTB43MuTSperm.bw
15ZBTB43WTSperm.bw

5VectorSSSi1.bw
6VectorSSSi2.bw
1VectorTKN1.bw
2VectorTKN2.bw
13ZBTB43SSSi1.bw
14ZBTB43SSSi2.bw
9ZBTB43TKN1.bw
10ZBTB43TKN2.bw

SSSi.bed
TKN.bed

ChIP-seq samples:
ChIP-seq IgG control15.5 dpc mouse prospermatogonia 100K cells
ChIP-seq IgG control15.5 dpc mouse prospermatogonia 415K cells
ChIP-seq Input 15.5 dpc mouse prospermatogonia 100K cells
ChIP-seq Input 15.5 dpc mouse prospermatogonia 491K cells
ChIP-seq ZBTB43 15.5 dpc mouse prospermatogonia 100K cells
ChIP-seq ZBTB43 15.5 dpc mouse prospermatogonia 491K cells

ChIP-seq raw files:
44IPIgG_L000_R1_001.fastq.gz
2IPIgG_L000_R1_001.fastq.gz
11In43_L000_R1_001.fastq.gz
6In43_L000_R1_001.fastq.gz
5IP43_L000_R1_001.fastq.gz
1IP43_L000_R1_001.fastq.gz
4IPIgG_L000_R2_001.fastq.gz
2IPIgG_L000_R2_001.fastq.gz
11In43_L000_R2_001.fastq.gz
6In43_L000_R2_001.fastq.gz
5IP43_L000_R2_001.fastq.gz
1IP43_L000_R2_001.fastq.gz

4IPIgG_filt_alns_keepdups.bw
2IPIgG_filt_alns_keepdups.bw
11In43_filt_alns_keepdups.bw
6In43_filt_alns_keepdups.bw
5IP43_filt_alns_keepdups.bw
1IP43_filt_alns_keepdups.bw

5IP43_vs_control_log2ratio.bw

	1IP43_vs_control_log2ratio.bw<br>input_as_control_S5IP43.bed<br>input_as_control_S1IP43.bed<br>5IP43_vs_control_log2ratio.bw<br>1IP43_vs_control_log2ratio.bw<br>igg_as_control_S5IP43.bed<br>igg_as_control_S1IP43.bed<br><br>igg_as_control_S5IP43.bed<br>igg_as_control_S1IP43.bed<br>input_as_control_S5IP43.bed<br>input_as_control_S1IP43.bed<br><br>MIRA-seq samples:<br>DNA methylation-MIRAseq of Zbtb43-/- adult sperm DNA replicate 1<br>DNA methylation-MIRAseq of Zbtb43-/- adult sperm DNA replicate 2<br>DNA methylation-MIRAseq of Zbtb43+/+ adult sperm DNA replicate 1<br>DNA methylation-MIRAseq of Zbtb43+/+ adult sperm DNA replicate 2<br><br>MIRA-seq raw files:<br>ZBTB4337_L000_R1_001.fastq.gz<br>ZBTB4338_L000_R1_001.fastq.gz<br>ZBTB4355_L000_R1_001.fastq.gz<br>ZBTB4356_L000_R1_001.fastq.gz<br><br>MIRA-seq processed files:<br>ZBTB43KO.bw<br>ZBTB43KOrep2.bw<br>ZBTB43WT.bw<br>ZBTB43WTrep2.bw
Genome browser session (e.g. UCSC)	N/A

Methodology

Replicates	Biological duplicates were used in the genomics experiment MIRA-seq, affinity-seq and ChIP-seq.
Sequencing depth	Affinity-seq: paired-end, 40M reads per sample.<br>MIRA-seq: single end, 40M reads per sample.<br>ChIP-seq: paired end, 40M reads per sample.
Antibodies	Rabbit anti-ZBTB43 antibody (Aviva Systems Biology Cat. No: ARP39048-P050, Lot QC19506-40743)<br>Rabbit anti-rabbit-IgG (Abcam Cat No: ab171870. Lot GR3228514-3)
Peak calling parameters	MIRA-seq and Affinity-seq analysis<br>Reads were aligned to the mm10 genome using bwa mem v0.7.17 with default parameters for MIRA-seq and Affinity-seq. Peaks were called for Affinity-seq samples using MACS2 callpeak v2.2.7.1 with default parameters, combining duplicate samples and using the corresponding MBP background samples as controls. Differentially methylated regions were called in MIRA-seq using MEDIPS with an extension size of 300 bp and window size of 100 bp. Differentially methylated windows with an adjusted P-value <0.05 were split by the log fold change direction. Adjacent windows were merged using bedtools merge v2.29.2 with the parameter '-d 1'.<br>ZBTB43 ChIP-seq analysis<br>Reads were trimmed using Trim Galore v0.6.0 (https://github.com/FelixKrueger/TrimGalore) then were aligned to the mm10 genome using bwa mem v0.7.17 51, using default parameters for both steps. Duplicate alignments were marked using SAMBLASTER v0.1.24 with the parameter, "--addMateTags". Alignments were filtered using samtools view v1.9 with the parameters, "-q 30 -F 2828 -f 2" and removing mitochondrial alignments. Peaks were called using MACS2 v 2.2.7.1 with the parameters, "-f BAMPE -g mm -q 0.05 --keep-dup 1" and the corresponding input or IgG samples as control. Peaks overlapping ENCODE blacklist v2 58 regions were removed using bedtools intersect v2.29.2 54 with the "-v" option.
Data quality	All peaks were called using a q-value (FDR) cutoff of 0.05.
Software	Softwares are described above. No novel software was generated.

Flow Cytometry

Plots

Confirm that:

☒ The axis labels state the marker and fluorochrome used (e.g. CD4-FITC).

☒ The axis scales are clearly visible. Include numbers along axes only for bottom left plot of group (a 'group' is an analysis of identical markers).

☒ All plots are contour plots with outliers or pseudocolor plots.

☒ A numerical value for number of cells or percentage (with statistics) is provided.

Methodology

Sample preparation	To obtain fetuses for wild type germ cell purification, C57BL6/J females were crossed with OG2 females (Oct4-EGFPTg/Tg; Zbtb43+/+). To obtain mutant fetuses, we crossed Zbtb43-/- mutant females with (Zbtb43-/-; Oct4-EGFPTg+) males. Testicles were dissected from the resulting male fetuses at different days of gestation, at 15.5 days post coitum (dpc) to 18.5 dpc, and were dissociated to single cell suspension using trypsin digestion and trituration as described earlier 16. 2-10 testes were digested in 150 µl 0.25% trypsin (including 0.5% BSA) at 36 °C for 15 min, tapping gently every 5 min. The trypsin reaction was stopped by adding 450 microliter 20% FBS in M2 medium.
Instrument	Beckman Coulter MoFlo Astrios sorter.
Software	Summit v 6.3
Cell population abundance	The cell purity was 95-99% based on re-sorting the EGFP+ cells.
Gating strategy	The healthy cell population was gated for positive population of singlets and then for positive population of EGFP expressing cells.

☒ Tick this box to confirm that a figure exemplifying the gating strategy is provided in the Supplementary Information.
