# Supplementary figures and images for: Z-DNA is remodelled by ZBTB43 in prospermatogonia to safeguard the germline genome and epigenome
Source: Nat Cell Biol. 2022 Jul 4;24(7):1141–53. doi: 10.1038/s41556-022-00941-9 (PMC9276527; doi:10.1038/s41556-022-00941-9)

Figure 2a

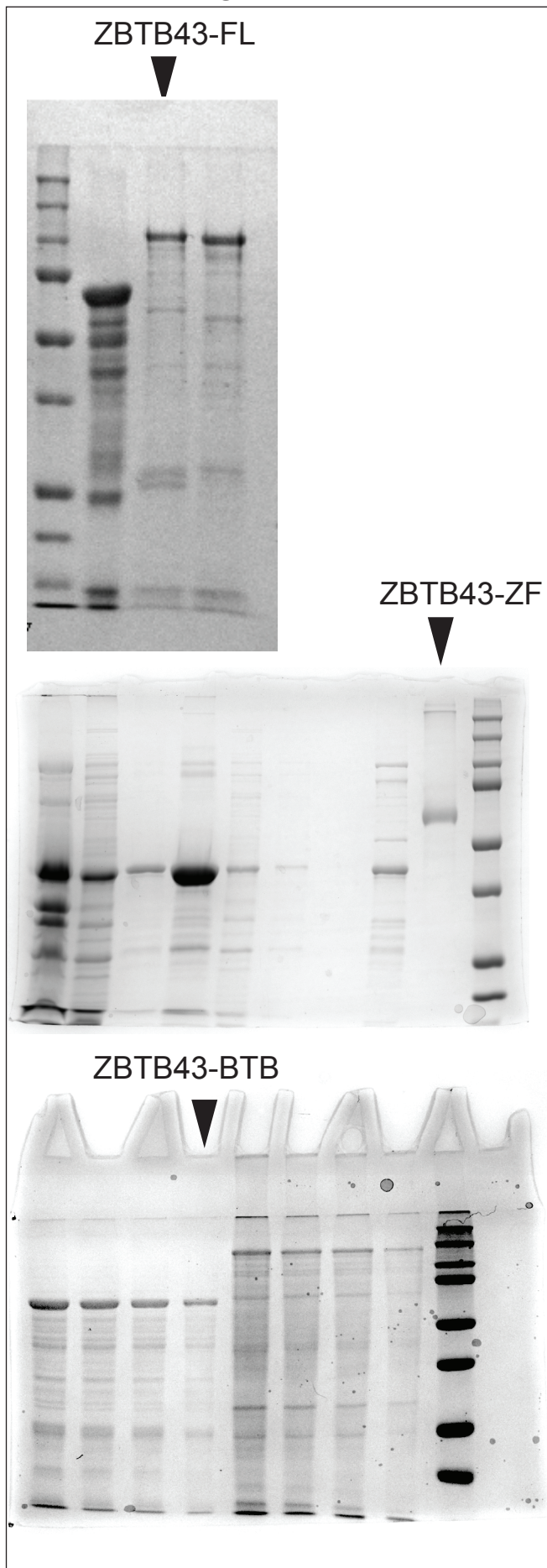

Figure 2g

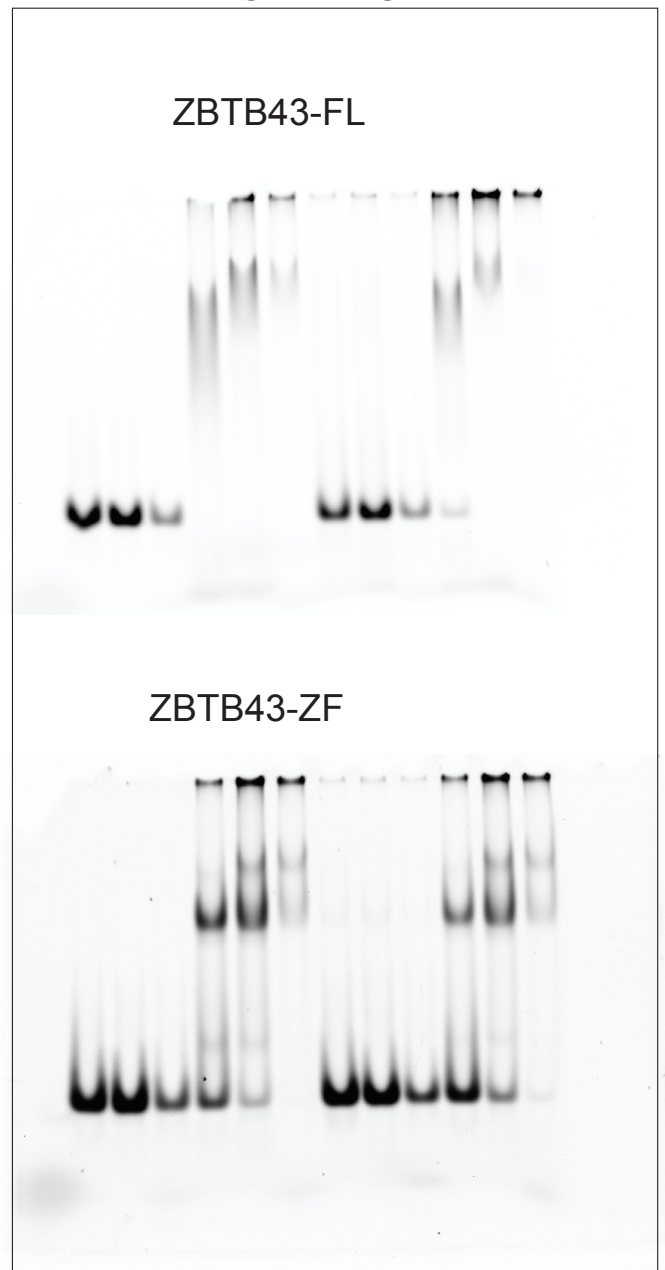

Fiaure 2h

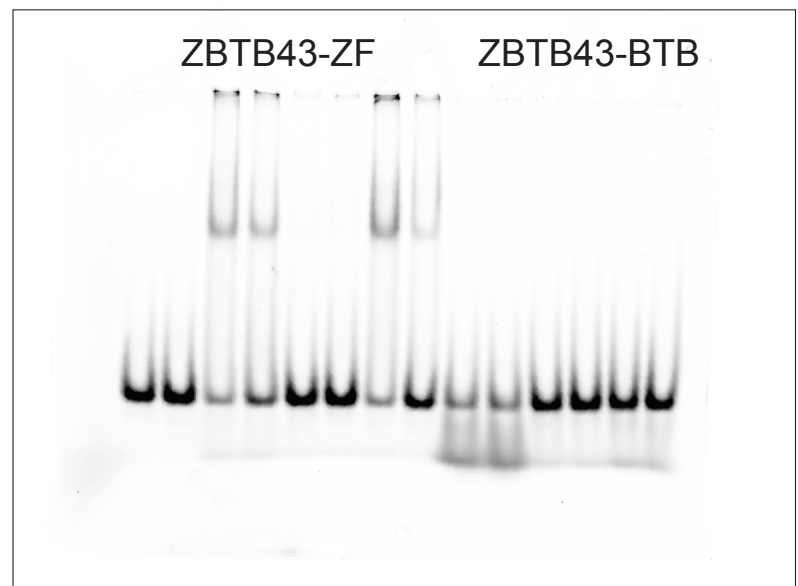

Supplement: Source Data Fig. 2 — Unprocessed blots and/or gels. [file 41556_2022_941_MOESM3_ESM.pdf]

Figure 4a

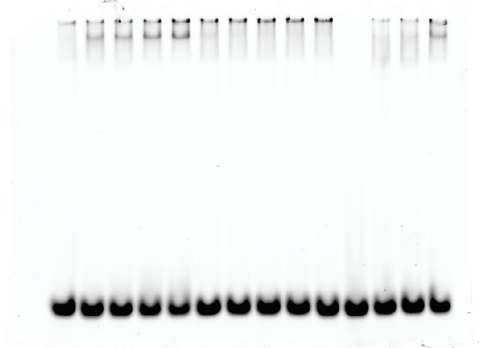

Figure 4e left

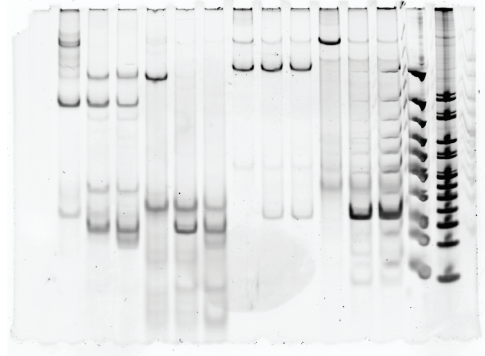

Figure 4f left

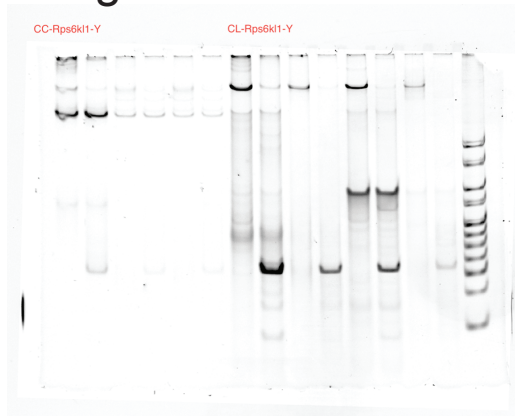

Figure 4f right, 4e right

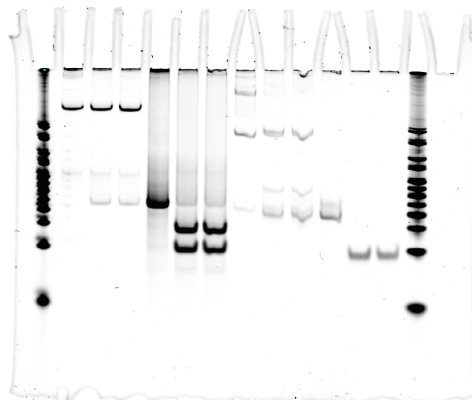

Figure 4g

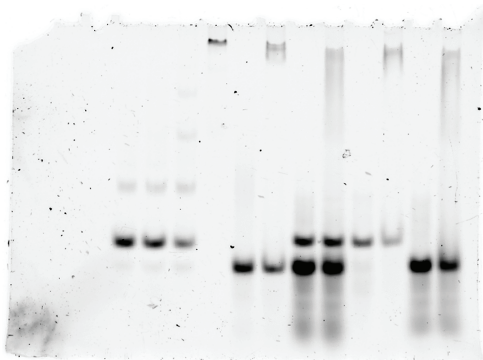

Figure 4g

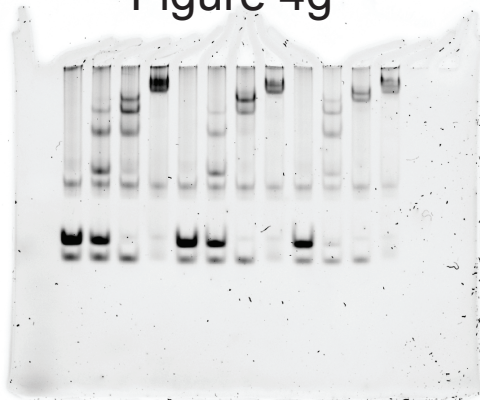

Figure 4h

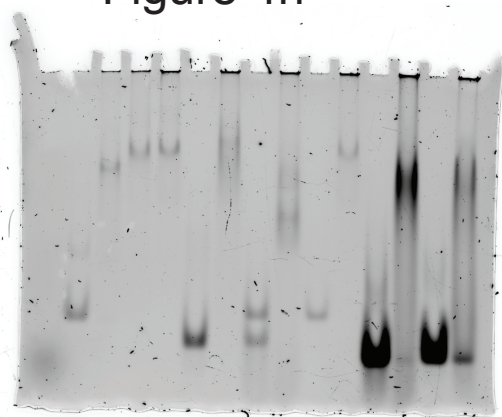

Figure 4h

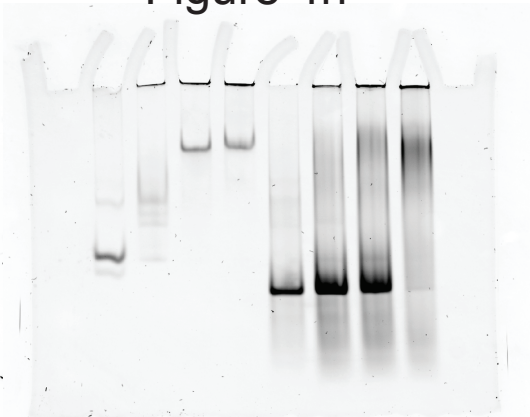

Supplement: Source Data Fig. 4 — Unprocessed blots and/or gels. [file 41556_2022_941_MOESM6_ESM.pdf]

Figure 5a

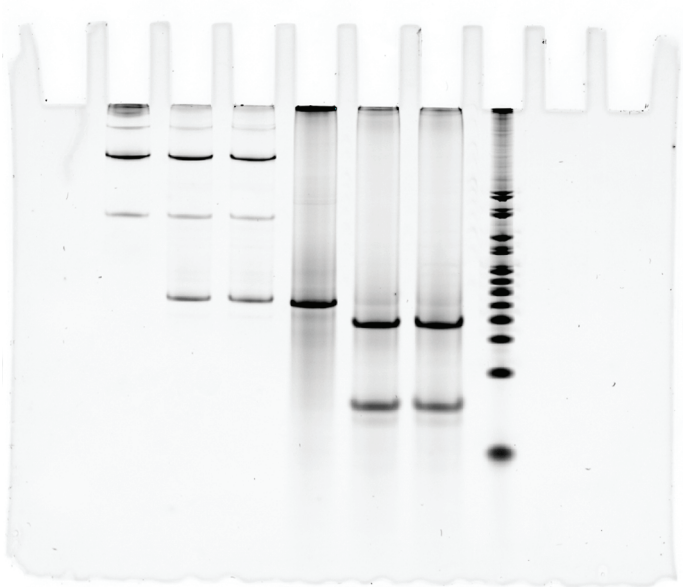

Figure 5d

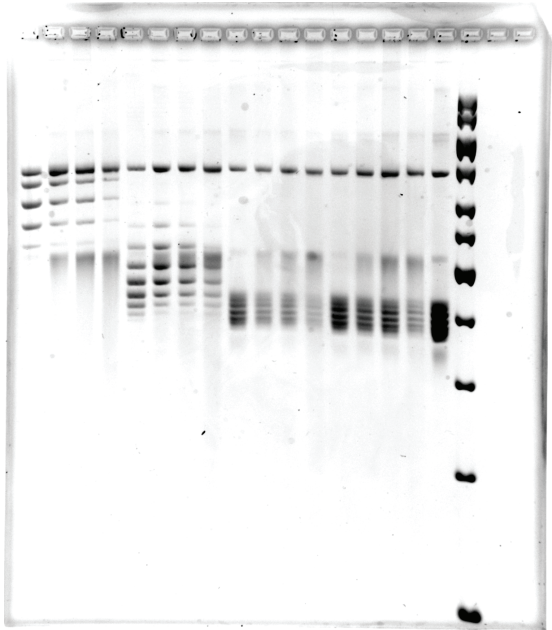

Figure 5b

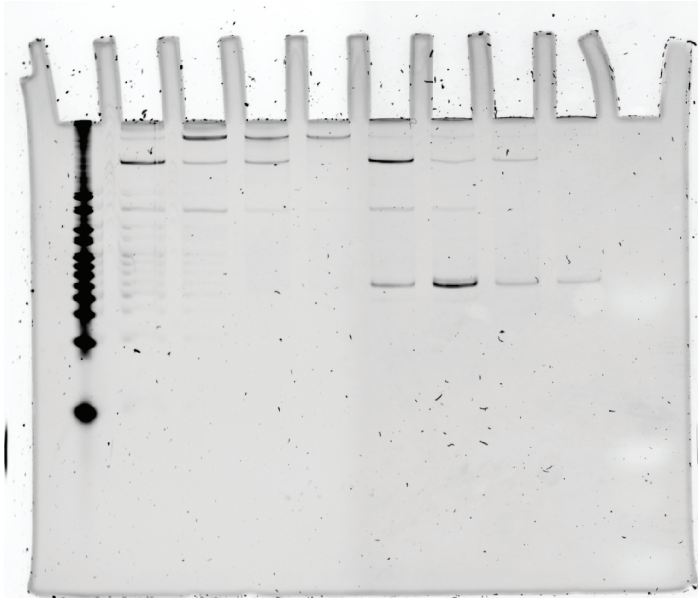

Figure 5c

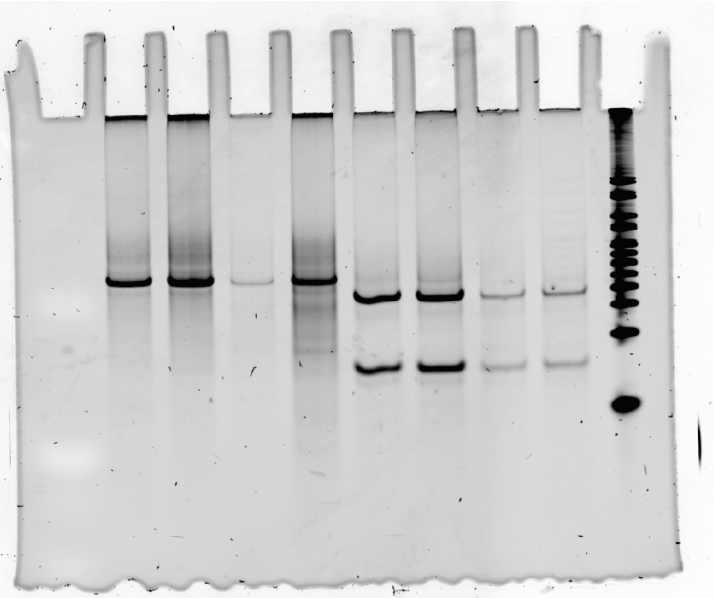

Supplement: Source Data Fig. 5 — Unprocessed blots and/or gels. [file 41556_2022_941_MOESM7_ESM.pdf]

Extended Figure 2b

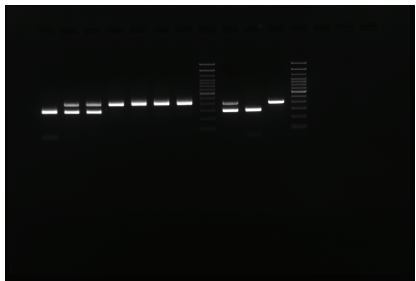

Extended Figure 2c

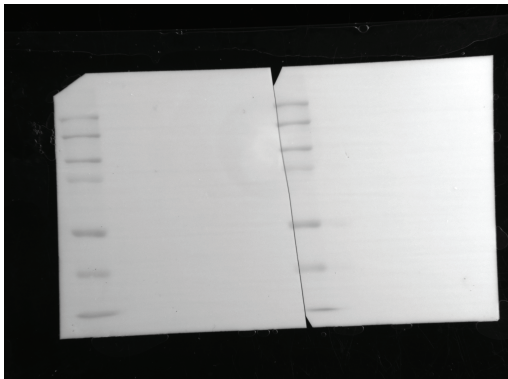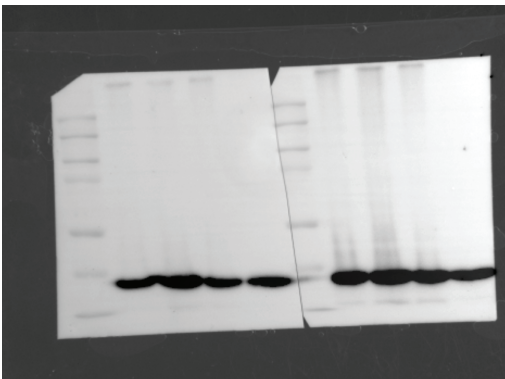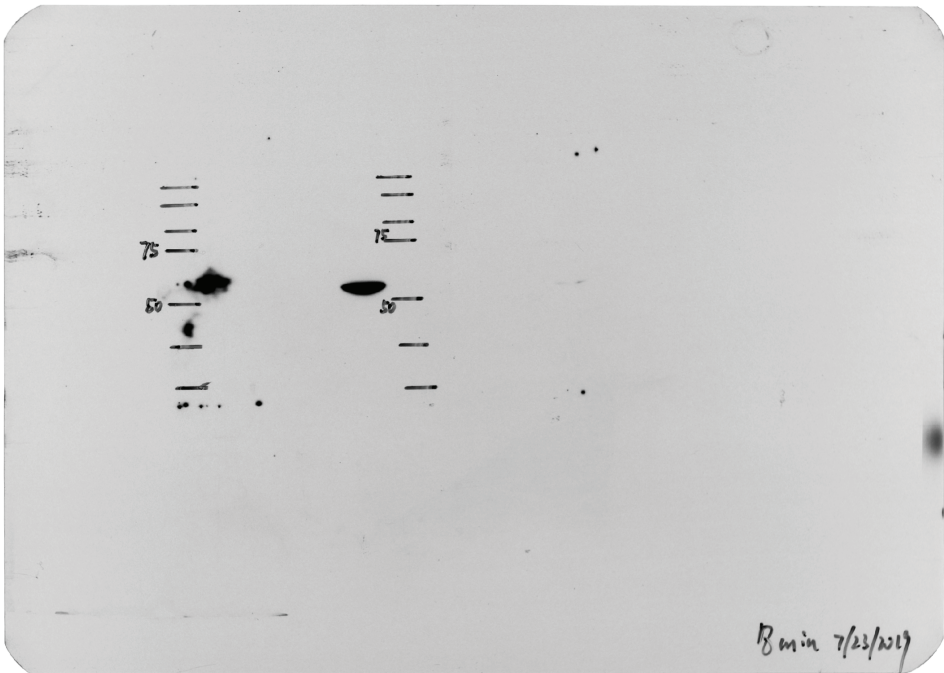

Extended Figure 2d

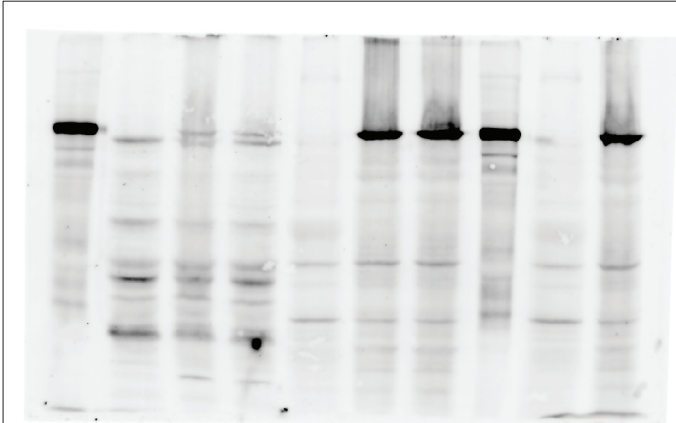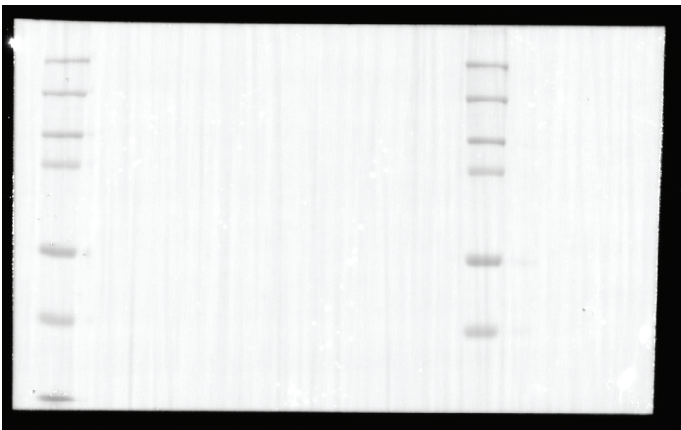

Supplement: Source Data Extended Data Fig. 2 — Unprocessed blots and/or gels. [file 41556_2022_941_MOESM9_ESM.pdf]
